# Slug Feeding Triggers Dynamic Metabolomic and Transcriptomic Responses Leading to Induced Resistance in *Solanum dulcamara*

**DOI:** 10.3389/fpls.2020.00803

**Published:** 2020-06-18

**Authors:** Onno W. Calf, Tobias Lortzing, Alexander Weinhold, Yvonne Poeschl, Janny L. Peters, Heidrun Huber, Anke Steppuhn, Nicole M. van Dam

**Affiliations:** ^1^Department of Molecular Interaction Ecology, Institute for Water and Wetland Research, Radboud University, Nijmegen, Netherlands; ^2^Department of Molecular Ecology, Institute of Biology, Free University of Berlin, Berlin, Germany; ^3^Department of Molecular Botany, Institute of Biology, University of Hohenheim, Stuttgart, Germany; ^4^German Centre for Integrative Biodiversity Research (iDiv) Halle-Jena-Leipzig, Leipzig, Germany; ^5^Institute of Biodiversity, Friedrich-Schiller University of Jena, Jena, Germany; ^6^Institute of Computer Science, Martin Luther University of Halle-Wittenberg, Halle (Saale), Germany; ^7^Department of Plant Systems Physiology, Institute for Water and Wetland Research, Radboud University, Nijmegen, Netherlands; ^8^Department of Experimental Plant Ecology, Institute for Water and Wetland Research, Radboud University, Nijmegen, Netherlands

**Keywords:** defense signaling, eco-metabolomics, LC-qToF-MS, microarray, plant–herbivore interaction, secondary metabolites

## Abstract

Induced plant responses to insect herbivores are well studied, but we know very little about responses to gastropod feeding. We aim to identify the temporal dynamics of signaling- and defense-related plant responses after slug feeding in relation to induced resistance. We exposed *Solanum dulcamara* plants to feeding by the gray field slug (GFS; *Deroceras reticulatum*) for different periods and tested disks of local and systemic leaves in preference assays. Induced responses were analyzed using metabolomics and transcriptomics. GFS feeding induced local and systemic responses. Slug feeding for 72 h more strongly affected the plant metabolome than 24 h feeding. It increased the levels of a glycoalkaloid (solasonine), phenolamides, anthocyanins, and trypsin protease inhibitors as well as polyphenol oxidase activity. Phytohormone and transcriptome analyses revealed that jasmonic acid, abscisic acid and salicylic acid signaling were activated. GFS feeding upregulated more genes than that it downregulated. The response directly after feeding was more than five times higher than after an additional 24 h without feeding. Our research showed that GFS, like most chewing insects, triggers anti-herbivore defenses by activating defense signaling pathways, resulting in increased resistance to further slug feeding. Slug herbivory may therefore impact other herbivores in the community.

## Introduction

In addition to producing constitutively expressed defenses, plants can increase their defenses upon herbivory (Agrawal, 1998; Walling, 2000; Wittstock and Gershenzon, 2002; Howe and Jander, 2008). Herbivore-induced responses are generally observed in local, damaged, as well as in systemic, undamaged, organs (van Dam and Raaijmakers, 2006; Chung et al., 2013). Deployment of inducible defense mechanisms, in concert with reorganizing primary metabolism, allows the plant to optimize resource investment under variable herbivore pressure (Schwachtje and Baldwin, 2008; Orians et al., 2011; Steinbrenner et al., 2011; Zhou et al., 2015). Induced responses show temporal dynamics; they commonly start with a build-up phase after the first damage and may decline after the herbivore has left (relaxation; de Vos et al., 2005; Mathur et al., 2011; Mason et al., 2017). The time-lag and attenuation of induced responses are cost-saving strategies to limit resource investment in defense when the chances of recurring feeding damage or the benefits of defense production are low (Karban, 2011; Underwood, 2012; Backmann et al., 2019). Moreover, plants may tolerate transient herbivory, e.g., by reallocating resources to unattacked organs for later regrowth (van der Meijden et al., 1988; Schwachtje et al., 2006; Orians et al., 2011). Tolerance and relaxation often act in synchrony with resistance mechanisms to attain an optimal response (Strauss and Agrawal, 1999; Stowe et al., 2000; Núñez-Farfán et al., 2007; Stout, 2013).

Induced defense responses can be tailored to specific herbivores (Agrawal, 2000; van Zandt and Agrawal, 2004; Chung and Felton, 2011). Plants perceive herbivory by chemical signals associated with feeding damage and with the herbivore itself, such as the herbivore’s saliva (Mithöfer and Boland, 2008; Bonaventure et al., 2011; Heil et al., 2012). These signals differ among herbivores, allowing plants to mount responses specific to the attacker (Voelckel and Baldwin, 2004; Basu et al., 2018). Herbivore-induced responses that follow lead to extensive molecular reprogramming (Leon et al., 2001; Maffei et al., 2007). This process is regulated by phytohormones, most importantly jasmonic acid (JA) and salicylic acid (SA; Erb et al., 2012). Cross-talk between the JA and SA signaling pathways results in specific responses to herbivores and pathogens (Lorenzo and Solano, 2005; Beckers and Spoel, 2006; Thaler et al., 2012; Schweiger et al., 2014). Other phytohormones, in particular abscisic acid (ABA) and ethylene (ET) are also involved in herbivore-induced defense responses (Nguyen et al., 2018).

While the mechanisms and consequences of insect-induced responses are intensively studied, similar studies addressing responses to gastropod feeding damage are scarce. It is likely that slug or snail feeding also elicits inducible defenses, because of the damage they inflict and the selective pressure they exert on plant populations (Strauss et al., 2009; Korell et al., 2016). The few studies analyzing gastropod-induced defenses show that they can increase defensive plant metabolites, including volatiles (Khan and Harborne, 1990; Viswanathan et al., 2005; Desurmont et al., 2016; Danner et al., 2017). Surprisingly, studies on the molecular regulation of gastropod-induced responses focussed on the effect of locomotion mucus, instead of actual feeding damage. These studies provided evidence that gastropod mucus applied to—artificially wounded—leaves can induce local and systemic responses (Orrock, 2013; Falk et al., 2014; Kästner et al., 2014; Meldau et al., 2014). Mucus-induced responses involve both JA- and SA-related defense signaling (Falk et al., 2014; Meldau et al., 2014). The mucus of the gray field slug (GFS; *Deroceras reticulatum*) contains SA (Kästner et al., 2014), which may suppress JA-dependent defense responses via negative cross-talk (Beckers and Spoel, 2006; Thaler et al., 2012; Schweiger et al., 2014). In addition to gastropod-specific elicitors in locomotion mucus, also the feeding mode of gastropods is different from that of chewing insects. Gastropods possess a radula, a tongue-like chitinous structure covered with minute teeth, which serves to scrape off leaf material. Artificial damage mimicking the damage inflicted by chewing herbivores followed by mucus application probably would not yield the full array of responses elicited by natural gastropod feeding (Lortzing et al., 2017). It is therefore essential to subject plants to real feeding damage to understand the full spectrum of responses induced by gastropod feeding.

We recently showed that 72 h feeding by GFS induces resistance to slugs in damaged leaves of bittersweet nightshade (*Solanum dulcamara*; Calf et al., 2019). The magnitude of the induced resistance varied among plant genotypes. However, slugs are voracious and opportunistic generalist herbivores, which leave their host plant after feeding at night. During the day, they commonly hide in the litter layer, after which they move to the same or another host-plant the following night (see Supplementary Video in Kästner et al., 2014). These temporal dynamics of slug-induced responses are of special interest, because plants may relax the induction of defenses if they experience only one feeding bout (Baldwin, 1996). This strategy would be reflected in the expression profiles of slug-induced responses. More specifically, we expect that long-term exposure to feeding could elicit stronger responses than short-term feeding, and that the induced response may relax after short-term feeding has stopped.

Here we experimentally assess the temporal and spatial dynamics of slug-induced responses in *S. dulcamara*, a common host for gastropods (Viswanathan et al., 2005; Lortzing et al., 2017). We specifically addressed the following questions: (1) Does GFS feeding induce GFS resistance in both local, damaged and systemic, undamaged leaves?; (2) What are the chemical, physiological and molecular mechanisms underlying induced responses and resistance to GFS feeding?; (3) Do induced responses and resistance depend on the duration of, and the time elapsed since, GFS feeding? In our first experiment, we exposed *S. dulcamara* plants to GFS feeding for 24 or 72 h and sampled local as well as systemic leaves at different time points thereafter. We used an eco-metabolomic approach (Peters et al., 2018) in which we combined untargeted metabolomic profiling of leaf samples with slug preference assays. This allowed us to assess locally and systemically induced metabolomic responses and their consequences for later arriving slugs. We further quantified the levels of polyphenol oxidase (PPO) activity and trypsin protease inhibitors (TPI). These are putative defenses that are commonly induced by insect herbivory on *S. dulcamara* (Viswanathan et al., 2005; Nguyen et al., 2016a; Lortzing et al., 2017). In addition to an increased production of defense compounds, herbivore feeding often reduces photosynthetic activity and induces leaf senescence (Wingler and Roitsch, 2008; Mellway et al., 2009; Zhu et al., 2017). For this reason, we measured chlorophyll and anthocyanin levels. In a second experiment, we exposed plants to GFS feeding for 24 h and performed microarray analyses on the transcriptome of local leaves directly at the end of the treatment period, as well as after an additional 24 h without feeding. In this experiment we also quantified the phytohormones JA, its bioactive isoleucine conjugate (JA-Ile), SA and ABA. This allowed us to assess the signaling events and induction processes directly after feeding by GFS and compare them to those after a 24 h relaxation period.

## Materials and Methods

### General Experimental Design

**FIGURE 1:**
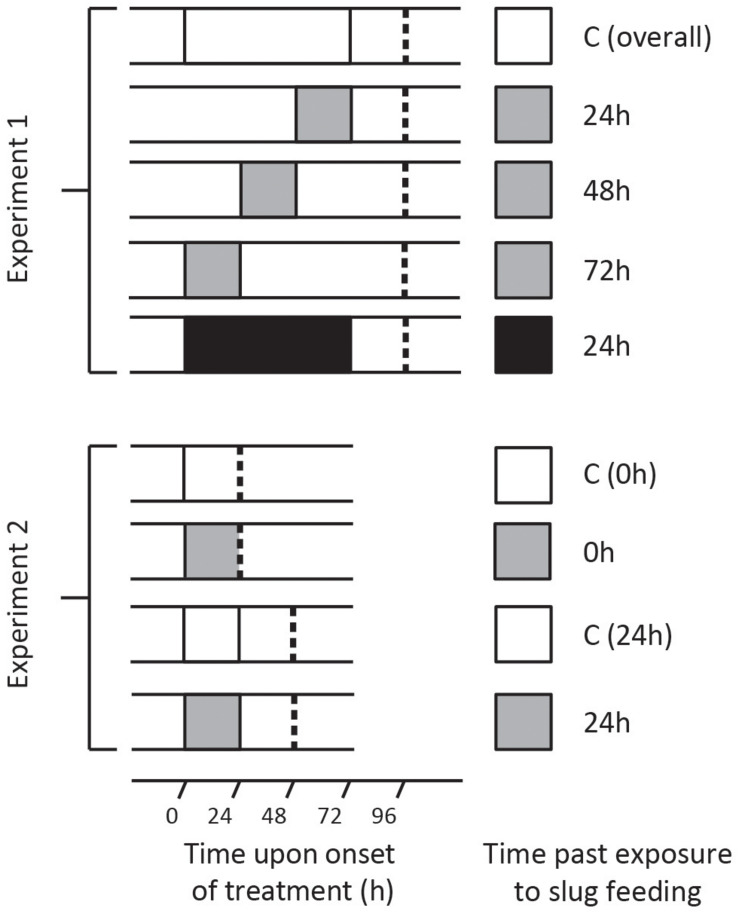
Treatment overview of two independent experiments in which *Solanum dulcamara* plants were exposed to an undamaged control treatment (C; white box) or to feeding by the gray field slug (GFS; *Deroceras reticulatum*) for 24 h (gray box) or 72 h (black box). Depending on the experiment and treatment, leaf harvest (dashed line) took place 0, 24, 48, or 72 h after the end of the treatment period.

Two independent experiments were performed to assess (1) induced resistance and metabolomic responses in local and systemic leaves, and (2) transcriptomic and phytohormone responses after feeding by GFS in *S. dulcamara*. To grow plants we used seeds collected in native *S. dulcamara* populations (locations provided below) following the procedure in Zhang et al. (2016). All plants and slugs were cultured under the same conditions as in Calf et al. (2018). Plants in the feeding treatments were fitted with clip cages on the tip of one or two leaves. This prevented that the whole leaf was consumed. Clip cages were left empty (control plants) or received one adult GFS. Depending on the experiment, GFS were left on the plant for 24 or 72 h (Figure 1). When harvesting, we assessed that all plants in the slug treatments showed substantial feeding damage (estimated by eye, >1 cm^2^).

### Experiment 1

Seeds of a single *S. dulcamara* population in the Netherlands (Goeree: 51°49′23.8′′N, 3°53′19.2′′E, provided by the Radboud University Genebank, Nijmegen, Netherlands) were used to grow plants. At the start of the experiment, the plants were 26 days old and 30–51 cm tall. Each plant was randomly assigned to one of five treatments (*n* = 6 plants/treatment; Figure 1, top half). Leaf number 10 and 11 from the apex were selected to receive GFS. Three groups of plants were exposed to GFS feeding for 24 h, and harvested 24, 48, or 72 h after the slugs had been removed (Figure 1, upper panel). An additional group of plants was exposed to 72 h GFS feeding and harvested 24 h after slug removal. Control plants were fitted with empty clip cages. These were left on the plant until the last clip cages were removed from the other plants (Figure 1). The leaves of the different treatment groups were harvested within 1 h (Figure 1).

The part of the leaf outside the clip cage was used to produce leaf disks using a cork-borer (1.5 cm Ø). One leaf disk was punched from each leaf (*n* = 12 disks/treatment/position). Leaf veins were avoided while punching out the disks. The remaining leaf material was sampled in liquid nitrogen and stored at −80°C until further processing. Disks and samples of leaf number 5 and 6 from the apex, which vascular tissue is fully connected to the treated leaves (Viswanathan and Thaler, 2004), were used to assess systemically induced responses.

#### Slug Preference Assays

The leaf disks were used in multiple choice slug preference assays to test the effect of GFS feeding on slug preference. One leaf disk of each treatment was offered to GFS in Petri dishes (*n* = 12; 5 disks per Petri dish) following the procedure described in Calf et al. (2019). Local and systemic leaves were tested separately.

#### Untargeted Metabolomic Profiling

An untargeted metabolomics approach was used to assess the effect of GFS feeding on the metabolomic profiles of *S. dulcamara* leaves. Leaf samples obtained from experiment 1 (*n* = 6 samples/treatment/leaf position) were extracted and analyzed by liquid chromatography – Time of Flight – Mass Spectrometry (LC-qToF-MS) following the method described in Calf et al. (2019). For methodological details see Document S1. Local and systemic leaf samples were analyzed in different sample runs. This approach resulted in a dataset with 142 and 170 mass signal clusters for the local and systemic sample sets, respectively. These groups potentially represent single metabolites. The signal with the highest intensity in the majority of the samples was selected to represent the respective metabolite in later analyses.

#### Targeted Analyses of Induced Responses

The levels of total proteins, PPO activity, TPI activity, chlorophyll and anthocyanin in local and systemic leaves (*n* = 6 samples/treatment/leaf position) were spectrophotometrically quantified from leaf extracts following methods by Bradford (1976) total proteins, Bode et al. (2013) PPO, Thaler et al. (1996) TPI, Wintermans and Demots (1965) chlorophyll, Nakata and Ohme-Takagi (2014) anthocyanin. See Supplementary Data Sheet S1 for details.

### Experiment 2

Four *S. dulcamara* seed batches were used to grow the plants; two batches originated from the Netherlands (Goeree: 51°49′23.8′′N, 3°53′19.2′′, registration nr. B24750010; Friesland: 52°58′36.2′′N, 5°30′59.4′′E, registration nr. B24750030, both provided by the Radboud University Genebank) and two from Germany (Erkner: 52°25′07.3′′N, 13°46′26.2′′E; Siethen: 52°16′53.7′′N, 13°11′18.7′′E). At the onset of the experiment, the plants were 25 days old and 27–45 cm tall. Plants were randomly assigned to one of four treatments (*n* = 16 plants/population/treatment, Figure 1, bottom half). Leaf number 10 from the apex was exposed to feeding by GFS for 24 h or fitted with an empty clip cage for the same time (undamaged control). Leaf harvest took place either directly (0 h) after removing the slugs or after an additional 24 h without GFS (Figure 1, lower panel). The total leaf area that was covered by the clip cage was collected for gene expression analyses. The leaf material outside the clip cage was sampled separately for phytohormone quantification. Thus we avoided contamination with locomotion mucus, which may contain SA (Kästner et al., 2014). All samples were flash frozen in liquid nitrogen and stored at −80°C until further processing.

#### Transcriptomic Analyses

The transcriptomic response upon GFS feeding was assessed using microarray analyses. Total RNA extraction and purification were performed on ground fresh leaf material using the RNeasy^®^ Plant Mini kit (Qiagen) and DNAse I (Fermentas, RNAse-free) following the manufacturer’s instructions. Quality and quantity of the RNA were estimated using a NanoDrop 1000 device (Thermo Fisher Scientific, Waltham, MA, United States) and 1.5% agarose gel electrophoresis. Equal quantities of total RNA from four individuals of a single plant population at each time point were pooled, resulting in four total RNA samples per treatment (control or induced). The RNA concentration, integrity and purity were assessed by electrophoretic analysis with the RNA 6000 Pico Kit using a 2100 Bioanalyzer (Agilent Technologies, Santa Clara, CA, United States)^1^. All samples had an RNA integrity number (RIN) between 6.6 and 7.2 and were hybridized on 8x60K Agilent microarrays. cDNA labeling, microarray hybridization, design were performed as described in Lortzing et al. (2017). The design and the experimental data of the microarray are available at the Gene Expression Omnibus of the National Centre for Biotechnology Information (NCBI; GEO Accession: GSE131208). RT-qPCR analyses were performed for technical validation of the microarrays (see Supplementary Data Sheet S1 and “Statistical Analyses” section).

#### Phytohormone Analyses

Phytohormones were quantified as described in Geuss et al. (2018) and see Supplementary Data Sheet S1 and Supplementary Table S1 for details on mass spectrometry. In brief, 100–110 mg of fresh leaf material (*n* = 4 samples/population/treatment) were ground and extracted two times with 1.0 ml ethyl acetate. Deuterated internal standards were included for salicylic acid (SA; OlChemIm Ltd., Olomouc, Czechia), abscisic acid (ABA; OlChemIm Ltd.), jasmonic acid (JA; Purity Compounds Standards GmbH, Cunnersdorf, Germany) and jasmonic acid-isoleucine (JA-Ile; HPC Standards GmbH). Extracts were vacuum-dried and re-eluted in 0.4 ml 70% MeOH containing 0.1% formic acid (v/v). Phytohormones were separated on a UPLC C18 column in a water^®^ and MeOH gradient (ACQUITY UPLC BEH-C18, 50 × 2.1 mm, particle size 1.7 μm), fragmented and detected in an ESI-MS/MS-qTOF detector (Synapt G2-S HDMS; Waters^®^, Milford, MA, United States) and quantified according to the respective internal standard.

### Statistical Analyses

Absolute leaf disk consumption (mm^2^) in the preference assays (experiment 1) was analyzed using non-parametric statistical methods from the R “stats” and “PMCMR” packages (Pohlert, 2014; R Core Team, 2016). Friedman’s rank sum test was applied followed by Conover’s *post hoc* test with Bonferroni correction for multiple comparisons to evaluate overall preference differences among treatments. The Petri dish number was used as grouping factor. The minimum consumption to accurately assess preference was 100 mm^2^ (11.7% of the offered leaf material). One replicate with less damage (local leaves) was excluded, leaving 11 replicates in this group.

The metabolomic datasets from experiment 1 were analyzed using the online R-based tool MetaboAnalyst 4.0 (Chong et al., 2018). The effect of GFS feeding on metabolomic profiles was assessed with discriminant analyses using orthogonal projection models to latent structures (OPLS-DA, Wiklund et al., 2008; Worley and Powers, 2013). These models separate metabolomic variation that is correlated with the treatment from random variation among samples. Individual OPLS-DA models were built to compare the generalized log-transformed (glog) intensity values of all metabolites in the control treatment to each of the GFS feeding treatments. Predictive significance of the models was assessed based on 1000 permutations using cross-validated predictive ability (Q^2^) as performance measure (Westerhuis et al., 2008; Worley and Powers, 2013). The correlation (*p*_corr_) of the response of each metabolite in relation to the first predictive component (p) from the model was used as a proxy for metabolite induction by GFS feeding. The explained variance by the predictive component [R^2^X_(p)_] was used as a proxy for the modeled effect size of the treatment. The local and systemic responses were tested separately. Metabolites that showed a high correlation with the model (*p*_corr_ > 0.7) and significant regulation according to Student’s *t*-test after Bonferroni correction for multiple comparisons (*P*_adj_ < 0.05), were selected as metabolites of interest. The relative levels of each metabolite of interest to the maximum mean value of this metabolite across all treatments was used to produce a heat map (%).

Microarray data were analyzed using the “limma” software packages from Bioconductor in “R” (Raj et al., 2006; R Core Team, 2016). Quantile normalized reads of 21261 out of 62970 contigs were included for data analysis after setting a critical level of quantification based on dark corner intensity (90% percentiles of non-labeled hairpin DNA probes) as well as removal of structural spots, repeatedly spotted probes, averaging repeatedly spotted contigs and selecting those contigs that were quantified in all samples of at least one treatment. Average fluorescence values of contigs were log_2_ transformed and visualized by principal component analyses (PCA) using the “prcomp” function. Data were fitted to a linear model using the “lmFit” function using dual contrasts (induced response at 0 or 24 h after exposure to GFS feeding versus the respective control). Contigs that showed an absolute log_2_-fold difference in expression >1 and significance (*P*_adj_) <0.05 after correction for false discovery rate according to the Benjamini–Hochberg method were considered significantly regulated. Contig expression levels in the microarray were validated based on Pearson’s correlation between fold-change in expression as estimated by microarray and RT-qPCR analyses for 10 genes related to primary and secondary metabolism (*PR1a*, *OPR3*, *PI1*, *KPI4*, *LOXD*, *PPOA*, *ERF4*, *PPO*, *NIM1* and *CS*, *R*^2^ = 0.97, Supplementary Figure S1 and Supplementary Data Sheet S1). Gene primers for qPCR were designed using the primer NCBI BLAST-tool^2^ (see Supplementary Table S2).

Functional descriptions of regulated contigs were obtained from the Sol Genomics Network (SGN^3^) and based on homology with tomato (D’Agostino et al., 2013). Gene ontology (GO) enrichment analyses for overrepresentation of molecular functions and biological processes were performed with the “TOPGO” package (Alexa and Rahnenfuhrer, 2010). GO annotation was based on Nguyen et al. (2016a). GO enrichment was assessed by comparing the distribution of the list of differentially expressed contigs to the distribution of all targets included in the data analysis. We used the “elim” algorithm, which corrects for the annotation of contigs in multiple parental GO terms (Alexa et al., 2006). Only GO terms that included a minimum of 50 annotated contigs were included for analyses. The *P*-values for the enrichment of each GO term are based on Fisher’s exact tests.

The effect of GFS feeding on levels of total proteins, PPO activity, TPI activity, anthocyanin and chlorophyll from (Experiment 1) as well as phytohormone levels (Experiment 2) were statistically tested using parametric statistical methods from the “stats,” “car” and “agricolae” software packages in “R” (Fox and Weisberg, 2011; R Core Team, 2016; de Mendiburu, 2017). Data were log_10_ or square root transformed when assumptions for homogeneity of variances among treatments and/or normal distribution of residuals were not met according to Levene’s and Shapiro–Wilk normality test, respectively. Treatment effects on metabolites and enzymes were tested using one-way ANOVA, followed by Tukey’s *post hoc* test for differences between treatments. Phytohormone levels were statistically tested using paired *t*-tests, including plant population of origin as paired factor.

## Results

### Induced Effect on GFS Feeding Preference (Experiment 1)

**FIGURE 2:**
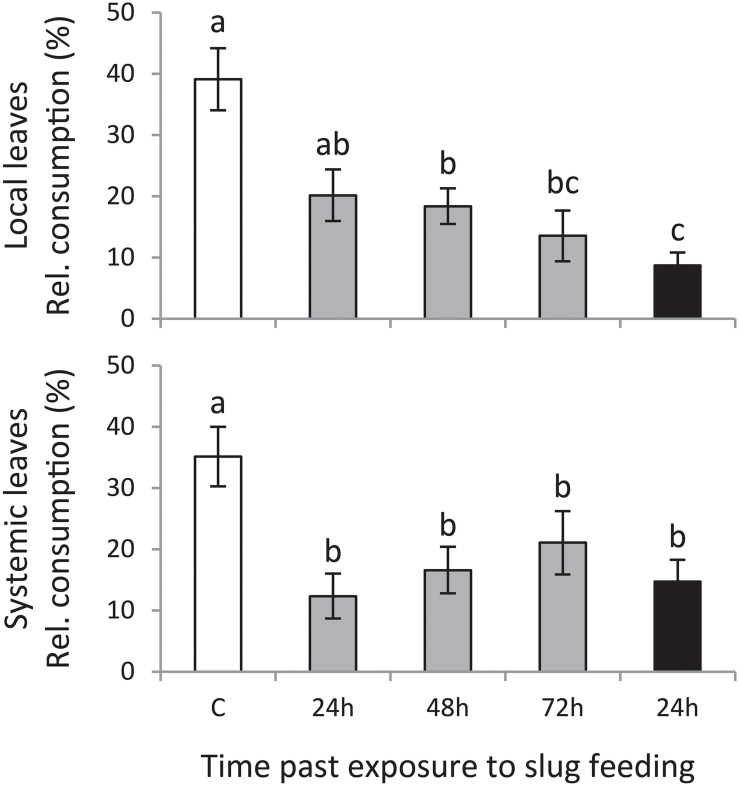
Mean consumed leaf area relative to the total consumed area (% ± SE, *n* = 12) on *Solanum dulcamara* leaf disks in preference assays with the gray field slug (GFS; *Deroceras reticulatum*). Slugs were offered leaf disks from plants that were exposed to an undamaged control treatment (C; white bar) or to feeding by GFS for 24 h (gray bars) or 72 h (black bar). Leaf disks were collected 24, 48, or 72 h after the end of the treatment period (see also Figure 1). Disks of either local or systemic leaves were offered in independent preference assays. Different letters over the bars indicate significant preference differences (*P* < 0.05) according to Conover *post hoc* test of Friedman ANOVA (χ^2^_local_ = 17.065, *P*_local_ = 0.002; χ^2^_systemic_ = 11.661, *P*_systemic_ = 0.022) after Bonferroni correction of *P*-values for multiple comparisons.

Slugs preferred to feed on leaf disks of plants that were left undamaged (control treatment) compared with those of plants exposed to GFS feeding (Figure 2). This preference was significant for both local and systemic leaves. A 24 h feeding period was sufficient to reduce slug preference on local leaves; 72 h continuous exposure to GFS resulted in the highest resistance. In contrast to the local leaves, systemically induced leaves were equally resistant, without a significant effect of the duration or timing of feeding.

### Metabolomic Profiling (Experiment 1)

OPLS-DA models were built to assess the effect of different temporal patterns of GFS feeding on leaf metabolomes (Table 1). All models, except one (systemic 24 h/24h; *Q*^2^: 48.8, *P* < 0.1, Table 1) had a significant predictive ability (*Q*^2^: 60.6–83.9%). The effect sizes [*R^2^X*_(p)_] of models testing the local response were always greater than the respective models testing the systemic response (Table 1). In both leaf positions, the effect size of the model increased with time after 24 h of exposure to GFS; 72 h continuous exposure to slugs elicited the strongest response.

**TABLE 1:** Results of OPLS-DA models comparing the metabolomic profiles (*n* = 6) of undamaged *Solanum dulcamara* leaves with those exposed to feeding by the gray field slug (GFS; *Deroceras reticulatum*) for 24 or 72 h.

OPLS-DA model	*Q*^2^	*R^2^X*_(p)_
***Local response***		
24 h/24 h	64.6*	14.9
24 h/48 h	66.3**	16.2
24 h/72 h	75.2**	19.9
72 h/24 h	83.9**	24.1
***Systemic response***		
24 h/24 h	48.8^+^	11.5
24 h/48 h	60.8*	13.1
24 h/72 h	60.6*	14.8
72 h/24 h	75.3**	16.0

Leaves were harvested at 24, 48, or 72 h past the end of the treatment period (see also Figure 1). Model codes indicate the duration of GFS feeding exposure and the time between the end of GFS exposure and harvest (duration/harvest). Separate models were built for local and systemic leaves. The cross-validated predictive ability (Q^2^) as well as explained percentage of variance by the predictive component [R^2^X_(p)_] are provided for each model. Symbols indicate significance of the cross-validated model based on 1000 permutations (**P < 0.01, *P < 0.05, ^+^P < 0.10).

**FIGURE 3:**
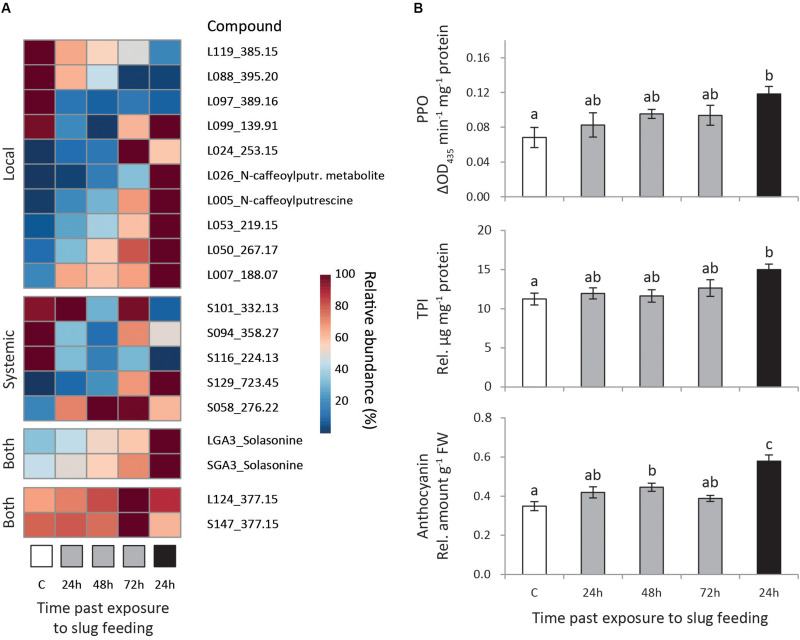
Induced responses in *Solanum dulcamara* leaves after feeding by the gray field slug (GFS, *Deroceras reticulatum*). Plants (*n* = 6) were left undamaged (control treatment: C, white) or exposed to feeding by GFS for 24 h (gray) or 72 h (black). Samples were collected 24, 48, or 72 h after the end of the treatment period (see also Figure 1). **(A)** Mean relative abundance of metabolites of interest, the levels of which were affected by GFS feeding in at least two treatments or in both local and systemic leaves within one treatment. The metabolite ID for local (L) and systemic (S) leaves is composed of a unique number and the quantified mass (*m/z*). **(B)** Mean relative levels (±SE) of polyphenol oxidase (PPO) activity, trypsin protease inhibitor (TPI) activity and total anthocyanins in local leaves. Treatment effects were significant according to one-way ANOVA (Table 2). Different letters over the bars indicate significant differences among treatments according to Tukey *post hoc* test (*P* < 0.05).

We selected metabolites correlating strongly with the predictive component of each model (*p*_corr_ > 0.7) and with significant regulation (*P*_adj_ < 0.05, Supplementary Table S3). Moreover, we focused on the induction pattern of those metabolites which levels were consistently regulated in at least two treatments, or in both local and systemic leaves within a treatment (Figure 3A).

Fifteen metabolites consistently changed in abundance in at least two treatments (Figure 3A; 10 local, 5 systemic). In local leaves, most selected metabolites (7 out of 10) gradually changed either up or downward with time after 24 h of GFS feeding. The strongest response was observed upon 72 h of continuous feeding (Figure 3A, Local). Two of these gradually changing metabolites reduced in abundance upon GFS feeding (L119 and L088), whereas five increased (L026, L005, L053, L050, and L007). Among the gradually increased metabolites there are two putatively identified phenolamides (see Calf et al., 2019) *N*-caffeoylputrescine and a related metabolite (L025 and L026 in Figure 3A, see also Supplementary Figure S2). In addition to the gradually regulated metabolites, one metabolite was strongly reduced in local leaves in all feeding treatments (L097, <20% of the control level). Two other metabolites showed the strongest response upon 24 h of exposure to GFS (L099 and L024). The changes in systemic leaves were more erratic. The gradual response that was observed for most locally regulated metabolites was only seen for two metabolites in systemic leaves (Figure 3A, systemic). One of these metabolites increased (S129) and the other (S116) decreased in abundance.

Two metabolites were found to be significantly regulated both in local and systemic leaves at one single time point only (Figure 3A, Both). One of these metabolites was the glycoalkaloid solasonine (LGA3/SGA3 in Figure 3A, identified by an authentic standard in Calf et al., 2018) which increased most strongly in abundance after 72 h continuous exposure to slug feeding (see also Supplementary Figure S2). In the 24 h feeding treatments, solasonine levels gradually increase with time after slug removal. The levels of an unidentified metabolite were also upregulated in both leaf positions (L124/S147, m/z 377.15 in Figure 3A), but only 72 h after 24 h of exposure to feeding. The relative increase of this metabolite was small, as constitutive levels (C) were high to start with (∼60% of the induced level).

### Targeted Analyses of Proteins and Compounds (Experiment 1)

We assessed the effect of GFS feeding on well-known insect inducible defensive proteins and compounds (Table 2 and Figure 3B). The levels of PPO activity, TPI activity and anthocyanins significantly increased in response to 72 h continuous exposure to feeding (Figure 3B), but only in local leaves (Table 2). A 24 h feeding period resulted in intermediate levels of these compounds. Total protein and chlorophyll levels were not affected by slug feeding (Table 2).

**TABLE 2:** One-way ANOVA table showing test results for the effect of feeding by the gray field slug (*Deroceras reticulatum*) on the levels of total proteins, polyphenol oxidase (PPO) activity, trypsin protease inhibitor (TPI) activity, anthocyanin and chlorophyll in local and systemic *Solanum dulcamara* leaves (*n* = 6, *df* = 4).

Parameter	*F*_Local_	*F*_Systemic_
Proteins mg g^–1^ FW	0.508	0.348
PPO rel. act. mg^–1^ protein	2.976*	0.164
TPI rel. μg mg^–1^ protein	3.297*	0.491
Anthocyanin rel. amount g^–1^ FW	11.850***	0.182
Chlorophyll rel. amount g^–1^ FW	0.366	1.754

Symbols indicate significance at the following levels: ***P < 0.001, *P < 0.05. Significant treatment effects are shown in Figure 3B.

### Transcriptomic Analyses (Experiment 2)

**FIGURE 4:**
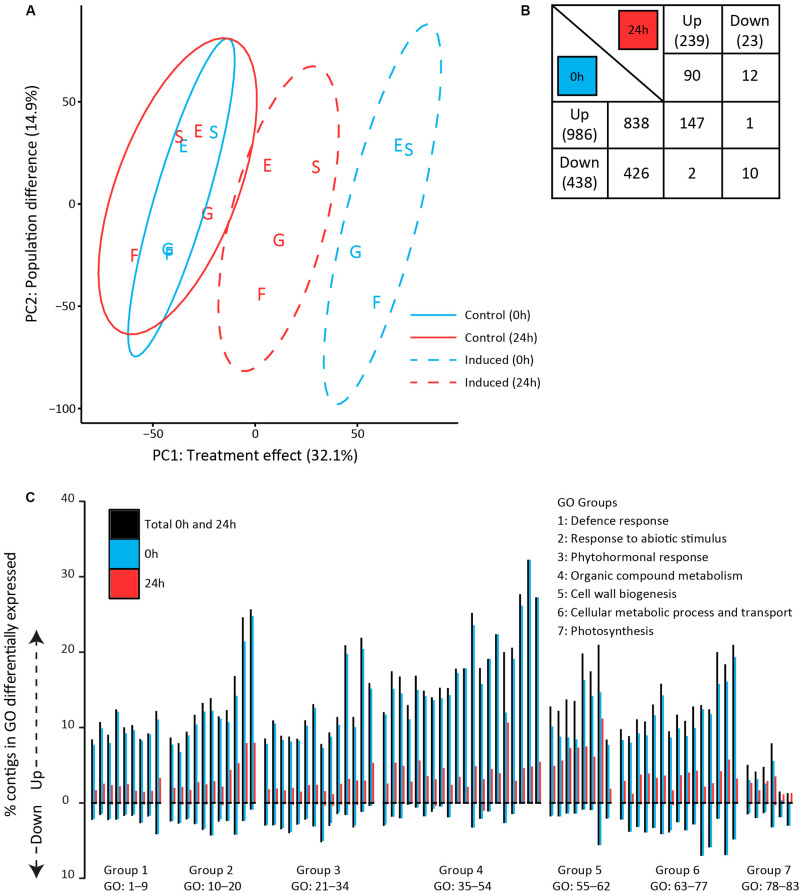
Transcriptomic profiles in *Solanum dulcamara* leaves upon 24 h of exposure to feeding by the gray field slug (*Deroceras reticulatum*). Samples were collected directly (0 h; blue) or 24 h (red) after the end of the treatment period (see also Figure 1). **(A)** Principal component analysis of overall transcriptomic differences among samples (*n* = 4/treatment/population). Ellipses show 95% confidence intervals. Letters indicate the plant population of origin; E, Erkner; S, Siethen; G, Goeree; F, Friesland. **(B)** Numbers of significantly up- and downregulated contigs that are unique for, or shared among harvest time points (fold change >2, *P*_adj_ < 0.05, *n* = 4). The total number of regulated contigs at each time point is given in brackets. **(C)** Percentage of contigs that was significantly up- or downregulated in 83 gene ontology (GO) terms. GO terms were grouped according to overall involvement in biological processes and sorted based on the number of contigs in each term (group numbers correspond to the numbers in Table 3).

To assess the early molecular signaling mechanisms preceding the observed metabolomic changes, we analyzed transcriptomes of plants that were sampled directly (0 h) after 24 h of exposure to feeding as well as after an additional 24 h without feeding (Figure 1, lower half). Principal component analyses were performed on the expression values of all quantified contigs (21261) to assess differences in the transcriptomic profiles (Figure 4A). The first principal component (PC1) captured the effect of GFS feeding, which accounted for 32.1% of the total variance among samples. Samples taken directly (0 h) after exposure to feeding separated further from the control samples than samples taken after an additional 24 h without feeding. The second principal component (PC2, 14.9%) revealed that transcriptomic profiles differed among plant populations. The German populations (Siethen and Erkner) separated from the Dutch populations (Goeree and Friesland).

A total of 1526 contigs (7.2% of the total set) was significantly up- or downregulated in response to feeding by GFS at either one of both time points (Figure 4B). The total number of regulated contigs directly (0 h) upon 24 h of exposure to feeding (1424) was >5 times greater than after an additional 24 h without feeding (262, Figure 4B). Overall, more contigs were upregulated than downregulated upon slug feeding. In the direct response treatment (0 h), the number of upregulated contigs (838) was twice as high as the number of downregulated contigs (426), whereas about an eightfold difference was observed after an additional 24 h without feeding (90 up, 12 down, Figure 4B). Two PPO enzymes (c2630 and c16387), five protease inhibitors (c460, c673, c1119, c4199, and c22651) and an anthocyanidin synthase (c10758) were among the 25 strongest regulated contigs at either one of both time points (Supplementary Table S4, 11–208 fold-change in expression).

Enrichment analyses of gene ontology terms helped to assess the biological processes that were overrepresented at either one or both time points. The 77 (out of 339) GO terms that were most significantly enriched upon slug feeding (*P*_adj_ < 0.0001) were selected and grouped according to the overall biological process they are involved in (group 1*–*6 in Table 3). In addition to the most strongly enriched terms, we selected six GO terms related to photosynthesis (group 7 in Table 3), which were previously found to be regulated upon insect feeding (Lortzing et al., 2017; Nguyen et al., 2018). The total number of up- and downregulated contigs in each term as well as the specific numbers at each time point were further evaluated (Figure 4C and Table 3). Most contigs in the selected GO terms were upregulated in response to slug feeding (Figure 4C). The numbers of regulated contigs were always higher directly (0 h) after 24 h of exposure to slug feeding than after an additional 24 h without feeding (Figure 4C; blue bar higher than red bar). Approximately 10*–*16% of all the contigs in terms related to defense responses (group 1) were significantly regulated. This group included various terms that involve responses to wounding, fungi, bacteria or nematodes (Table 3). The many responses related to abiotic stimuli (group 2) indicate that there is large overlap in responses to biotic and abiotic stressors. Various terms related to defense-signaling phytohormones (group 3) were regulated upon slug feeding. These included JA (4 terms), SA (3 terms), ABA (2 terms) as well as single terms related to auxin, karrikin, gibberellin, ethylene and brassinosteroids (Table 3). The highest percentage of phytohormone-related contigs was regulated for JA metabolic (GO:31) and biosynthetic processes (GO:33, both about 23%, Figure 4C and Table 3). The overall largest percentage of regulated contigs was observed for terms involved in organic compound metabolism (up to 35%, group 4). This group includes biosynthetic processes for many defense-related compounds, such as flavonoids, phenylpropanoids, coumarins and alkaloids. Also terms related to primary metabolism, such as phenylalanine and tyrosine metabolism, were enriched (group 4). Genes involved in cell wall biogenesis (group 5) are likely involved in wound healing. The upregulation of many basic cellular metabolic and transport processes (group 6) indicates extensive reprogramming of the plants’ primary metabolism upon slug feeding. In contrast, only relatively few contigs involved in photosynthesis were regulated (group 7). Most genes in this category were upregulated, but only term 81 (chlorophyll biosynthetic process) was significantly enriched (*P*_adj_ = 0.007; Figure 4C).

**TABLE 3:** Gene ontology (GO) terms that were enriched strongest upon 24 h of exposure to feeding by the gray field slug (*Deroceras reticulatum*) as well as six terms that involve photosynthesis (Group 7).

#	GO.ID	GO term description	Contigs in term	*n* regulated contigs		
				Total	0 h	24 h
***Group 1: Defence response***						
1	GO:0006952	Defense response	4146	440	407	76
2	GO:0009620	Response to fungus	1567	192	178	42
3	GO:0042742	Defense response to bacterium	1361	152	136	35
4	GO:0009611	Response to wounding	1314	190	186	31
5	GO:0050832	Defense response to fungus	1106	129	119	30
6	GO:0009627	Systemic acquired resistance	1056	126	118	19
7	GO:0010200	Response to chitin	968	107	105	16
8	GO:0010363	Regulation of hypersensitive response	889	97	96	15
9	GO:0009624	Response to nematode	361	58	54	12
***Group 2: Response to abiotic stimulus***						
10	GO:0009651	Response to salt stress	2286	252	231	47
11	GO:0009409	Response to cold	1714	182	161	40
12	GO:0006979	Response to oxidative stress	1462	168	160	28
13	GO:0009414	Response to water deprivation	1421	204	186	40
14	GO:0042538	Hyperosmotic salinity response	528	87	81	14
15	GO:0010167	Response to nitrate	417	75	68	12
16	GO:0010224	Response to UV-B	322	44	43	8
17	GO:0010583	Response to cyclopentenone	252	37	33	11
18	GO:0010106	Cellular response to iron ion starvation	190	40	35	10
19	GO:0009269	Response to desiccation	126	34	30	10
20	GO:0071456	Cellular response to hypoxia	113	30	29	9
***Group 3: Phytohormonal response***						
21	GO:0009737	Response to abscisic acid	1932	221	207	36
22	GO:0009753	Response to jasmonic acid	1243	171	166	26
23	GO:0009751	Response to salicylic acid	1202	147	139	22
24	GO:0009733	Response to auxin	1045	133	125	24
25	GO:0009738	Abscisic acid-activated signaling pathway	798	90	88	13
26	GO:0009867	Jasmonic acid mediated signaling pathway	721	94	89	18
27	GO:0080167	Response to karrikin	580	94	91	15
28	GO:0009739	Response to gibberellins	575	75	71	11
29	GO:0009862	SA-mediated systemic acquired resistance	524	64	60	8
30	GO:0009696	Salicylic acid metabolic process	474	60	55	13
31	GO:0009694	Jasmonic acid metabolic process	440	99	94	15
32	GO:0009873	Ethylene-activated signaling pathway	368	53	48	12
33	GO:0009695	Jasmonic acid biosynthetic process	338	78	73	10
34	GO:0016132	Brassinosteroid biosynthetic process	264	43	41	14
***Group 4: Organic compound metabolism***						
35	GO:0009813	Flavonoid biosynthetic process	623	93	91	17
36	GO:0009698	Phenylpropanoid metabolic process	601	116	102	32
37	GO:0009699	Phenylpropanoid biosynthetic process	489	92	81	24
38	GO:0006633	Fatty acid biosynthetic process	424	56	48	12
39	GO:0016126	Sterol biosynthetic process	320	56	50	18
40	GO:0009805	Coumarin biosynthetic process	282	47	45	10
41	GO:0009963	Positive regulation of flavonoid biosynthesis	257	38	37	9
42	GO:0006084	Acetyl-CoA metabolic process	216	34	31	10
43	GO:0042398	Cellular modified amino acid biosynthesis	210	36	34	5
44	GO:0006570	Tyrosine metabolic process	174	31	30	6
45	GO:0006558	L-phenylalanine metabolic process	140	25	25	3
46	GO:0006598	Polyamine catabolic process	123	35	33	6
47	GO:0006639	Acylglycerol metabolic process	95	19	17	4
48	GO:0018874	Benzoate metabolic process	89	18	18	4
49	GO:0042343	Indole glucosinolate metabolic process	76	17	17	3
50	GO:0019852	L-ascorbic acid metabolic process	75	17	11	8
51	GO:0071616	Acyl-CoA biosynthetic process	68	15	14	2
52	GO:0009821	Alkaloid biosynthetic process	65	18	17	3
53	GO:0016104	Triterpenoid biosynthetic process	62	20	20	3
54	GO:0019745	Pentacyclic triterpenoid biosynthetic process	55	15	15	3
***Group 5: Cell wall biogenesis***						
55	GO:0042546	Cell wall biogenesis	899	130	106	45
56	GO:0010383	Cell wall polysaccharide metabolic process	729	102	77	41
57	GO:0045492	Xylan biosynthetic process	357	54	36	26
58	GO:0010413	Glucuronoxylan metabolic process	355	53	35	26
59	GO:0009808	Lignin metabolic process	227	47	39	17
60	GO:0009809	Lignin biosynthetic process	212	39	32	13
61	GO:0009834	Plant-type secondary cell wall biogenesis	143	38	29	16
***Group 6: Cellular metabolic process and transport***						
62	GO:0055114	Oxidation-reduction process	2068	216	201	41
63	GO:0005982	Starch metabolic process	817	98	86	24
64	GO:0009744	Response to sucrose	575	73	68	7
65	GO:0000041	Transition metal ion transport	532	76	66	21
66	GO:0009750	Response to fructose	435	64	56	17
67	GO:0005985	Sucrose metabolic process	421	69	63	14
68	GO:0015706	Nitrate transport	386	76	70	14
69	GO:0010310	Regulation of hydrogen peroxide metabolism	358	47	44	7
70	GO:0006826	Iron ion transport	273	39	34	10
71	GO:0042744	Hydrogen peroxide catabolic process	248	36	31	10
72	GO:0000272	Polysaccharide catabolic process	211	33	27	9
73	GO:0006857	Oligopeptide transport	185	36	35	4
74	GO:0015824	Proline transport	153	28	27	4
75	GO:0009225	Nucleotide-sugar metabolic process	95	21	17	4
76	GO:0015837	Amine transport	87	22	20	5
77	GO:0048359	Mucilage metabolism (seed coat development)	62	16	15	2
***Group 7: Photosynthesis***						
78	GO:0015979	Photosynthesis	793	51	35	22
79	GO:0009658	Chloroplast organization	601	37	31	10
80	GO:0019684	Photosynthesis, light reaction	585	35	22	18
81	GO:0015995	Chlorophyll biosynthetic process	341	38**	30	13
82	GO:0010207	Photosystem II assembly	264	9	6	3
83	GO:0009902	Chloroplast relocation	232	10	7	3

GO term numbers (#) correspond to the number in Figure 4C. GO terms were grouped according to overall involvement in biological processes and sorted based on the number of contigs in each term. The number (n) of regulated contigs is given for the total response as well as separate for two time points (0 and 24 h) after the end of the treatment. All terms in group 1–6 were significantly regulated (P_adj_ < 0.0001) according to Fisher’s exact test after correction for false discovery rate using the Bonferroni method. Symbols in group 7 indicate significance on the following level: **P_adj_ < 0.01.

### Phytohormone Analyses (Experiment 2)

**FIGURE 5:**
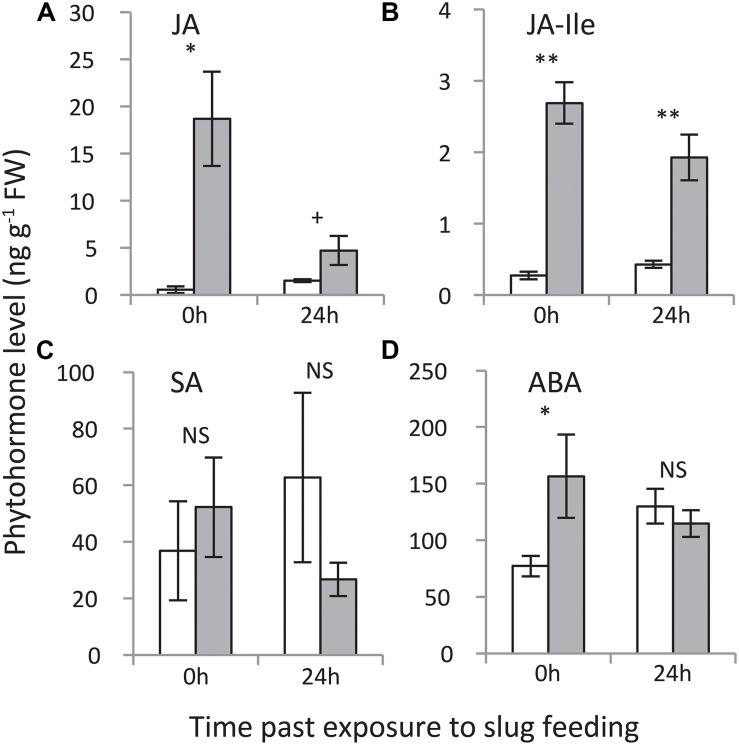
Levels of phytohormones in *Solanum dulcamara* leaves upon 24 h of exposure to feeding by the gray field slug (*Deroceras reticulatum*). **(A)** jasmonic acid, **(B)** jasmonic acid-isoleucine, **(C)** salicylic acid, and **(D)** abscisic acid. Samples were collected directly (0 h) or 24 h after the end of the treatment period (see also Figure 1). Bars represent mean levels (±SE, *n* = 4) in undamaged control (white bars) and damaged leaves (gray bars). The symbols indicate significant differences between treatments on a single time point according to Welch’s *t*-test on the following levels: **P < 0.01, *P < 0.05, ^+^*P* < 0.1.

Jasmonic acid as well as the levels of its bioactive isoleucine conjugate (JA-Ile) were elevated at both time points, with the strongest increase measured directly (0 h) after 24 h of exposure to GFS feeding (Figure 5). Salicylic acid levels were not affected, whereas ABA levels were elevated directly (0 h) after 24 h of exposure to slug feeding, but not after an additional 24 h without feeding.

## Discussion

Our study revealed that feeding by gray field slugs (GFS) elicits jasmonic acid-based defense responses in *S. dulcamara* resulting in local and systemic induced resistance to conspecifics (Karban and Baldwin, 1997). Twenty-four hours of slug feeding sufficed to increase the levels of JA, JA-Ile and ABA, as well as significant metabolomic and transcriptomic changes. GFS feeding triggered transcription of genes related to diverse pathways, including JA and SA phytohormone signaling. Plants responded to GFS feeding by increasing commonly known defenses, such as solasonin, TPIs, PPOs, phenolamides and anthocyanins. This indicates that GFS feeding induces similar signaling pathways and defense metabolites as chewing insects. Other than insect feeding, slug herbivory did not affect chlorophyll levels or downregulate the transcription of photosynthesis-related genes. Whereas the magnitude of the transcriptomic response was reduced within 24 h after the slugs were removed, the metabolomic response kept increasing with time. The longest feeding period of 72 h resulted in the strongest metabolomic response and the highest resistance level. Taken together, this suggests that *S. dulcamara* limits resource investments in GFS-induced defenses while conserving primary metabolism in the absence of further slug feeding.

Slug feeding triggered several phytohormones involved in defense signaling; JA, JA-Ile and ABA levels were significantly elevated upon slug feeding, which was also reflected by the transcriptomic response in the respective GO categories. Remarkably, SA levels were unaffected, whereas genes involved in SA metabolism and signaling were upregulated. This may be explained by the earlier finding that GFS locomotion mucus contains SA (Kästner et al., 2014). In this study, application of slug mucus to Arabidopsis thaliana leaves induced the local expression of PR1, a marker gene for SA defense signaling (Kästner et al., 2014). SA and JA both are involved in responses to herbivore feeding whereby they often act in negative cross-talk (Beckers and Spoel, 2006; Thaler et al., 2012; Schweiger et al., 2014). This triggered the speculation that GFS may benefit from the SA in the mucus, because it suppresses JA herbivore defenses (Kästner et al., 2014). We did not test whether the locomotion mucus of the field-collected slugs in our study contained SA, but the expression of a *S. dulcamara* PR1 homolog was unaffected by GFS feeding (c374 in Supplementary Figure S1). Moreover, JA and JA-Ile levels, as well as jasmonic acid related gene expression, increased strongly upon slug feeding in *S. dulcamara*. If the mucus of our GFS would have contained SA, the levels were not sufficient to antagonize the JA-related induced responses or prevent induced resistance to conspecific slugs.

Both in the transcriptome and the metabolome we identified different responses that may cause slug resistance. Glycoalkaloids likely play a key role in resistance to GFS. Solasonine levels were significantly higher in both local and systemic leaves upon 72 h of exposure to slug feeding, and leaf disks of these plants were also the least preferred by the GFS. Moreover, we previously showed that constitutive resistance to GFS in *S. dulcamara* is associated with high levels of glycoalkaloids (Calf et al., 2018). Taken together, this suggests that glycoalkaloids cause both constitutive and induced resistance to slugs. In a common garden experiment with different *S. dulcamara* glycoalkaloid chemotypes we found that gastropods and specialist flea beetles show opposite feeding preferences for glycoalkaloid chemotypes (Calf et al., 2019). This implies that slug-induced increases in glycoalkaloid levels may have opposing effects on the many members of the natural *S. dulcamara* herbivore community (Calf and van Dam, 2012).

In addition to glycoalkaloids, we found several other induced defenses that possibly affect slug preference. Genes related to TPI and PPO activity were among the most strongly regulated genes after 24 h exposure to GFS feeding. This is in line with their increased activity in local leaves, in particular after 72 h continuous exposure to slugs. Previous studies found that PI levels and PPO activity as well as transcription of genes coding for these proteins increased upon insect feeding or oviposition on *S. dulcamara* (Boege and Marquis, 2005; Nguyen et al., 2016b; Geuss et al., 2017; Lortzing et al., 2017). Increases in PI and PPO levels correlated with increased resistance to several insect herbivores and reduced egg hatching (Geuss et al., 2018; Nguyen et al., 2018). Increased PI levels can reduce slug feeding. Leaf damage by slug feeding was reduced by 50% in Arabidopsis plants genetically modified to overexpress a cysteine protease inhibitor from rize, oryzacystatin (Walker et al., 1999). However, it is not fully clear yet whether TPIs, which are serine protease inhibitors, also confer resistance to GFS, which mainly produces cysteine proteases in its digestive glands (Walker et al., 1998). Although cystein PI activity was not measured from our leaf extracts, a contig for cystein PI8 (c4199 in Supplementary Table S4) was among the 25 most strongly regulated contigs. Because TPIs are effective resistance factors to insect herbivores, in particular lepidopteran larvae (Glawe et al., 2003; Bode et al., 2013; Zhu-Salzman and Zeng, 2015) it is likely that slug-induced responses affect other herbivores in the community.

The transcriptomic and metabolomic analyses showed that anthocyanins and phenolamides were also induced. Each of these metabolites, or genes coding for their biosynthesis, were previously found to be induced upon insect feeding or pathogen infection as well as primed by oviposition (Nguyen et al., 2016b; Geuss et al., 2018). Anthocyanins have multiple roles in plants, including resistance to insect herbivores (Gould, 2004). Phenolamines, such as caffeoylputrescine, are intermediates in lignin biosynthesis (Gaquerel et al., 2014). The genes involved in this pathway dynamically respond to biotic stress such as herbivore feeding. Increased resistance may be conferred by one of the many compounds in the pathway or by increased leaf toughness due to an increase in lignin concentration. The strong transcriptomic response in the GO related to phenylpropanoids (for example GO:0009698 phenylpropanoid metabolic process; GO:0009805 coumarin biosynthetic process and GO:0009808 lignin metabolic process) indicates that slug and insect feeding trigger similarly strong responses in the phenolamide pathway. Whether or not this is indeed a functional response leading to reduced herbivore damage, as suggested by Gaquerel et al. (2014) remains to be determined.

Interestingly, we observed that, overall, slug feeding upregulated transcriptional responses in the primary metabolism. The many transcriptomic responses related to abiotic stimuli indicate that there is large overlap in responses to biotic and abiotic stress factors. Similar results were reported in an experimental analysis on interactive responses to drought, flooding and insect feeding in *S. dulcamara* (Nguyen et al., 2018). Such cross-talks between signaling pathways allow the plant to orchestrate both resource acquisition and defense allocation in complex natural environments (Nguyen et al., 2016b). Chewing insect herbivore damage, for example, can downregulate photosynthesis-related processes in *S. dulcamara* (Lortzing et al., 2017; Nguyen et al., 2018) and other plant species (Zangerl et al., 2002; Tang et al., 2006; Lawrence et al., 2008; Bilgin et al., 2010). However, local and systemic herbivore-induced responses on the level of photosynthetic pathways cover the whole range from positive to negative gene regulation (Zhou et al., 2015). This led to the contrasting hypotheses that upregulation of photosynthetic capacity is a way to increase carbon for defense production, whereas downregulation of the costly photosynthetic machinery would free up resources for defense related pathways (Zhou et al., 2015). In our current study, only a few genes involved in photosynthesis were significantly regulated and chlorophyll levels did not change. These results suggest that the photosynthetic capacity of the leaves was maintained. Taken together, GFS-induced resistance in *S. dulcamara* may not come at a cost in terms of reduced primary metabolism (Zhou et al., 2015).

The observed temporal and spatial patterns suggest that *S. dulcamara* may optimize its response to GFS feeding. The strength of the induced response depended both on the period the slug was on the plant and the time that past after the slug was removed. The transcriptomic response was strongly relaxed in the absence of further damage. This is in line with our observation that the metabolomic response after 72 h of feeding is stronger than after a 24 h feeding bout. In contrast to the transcriptomic response, the metabolomic response to 24 h feeding gained in strength with time after slug removal. The observed metabolomic induction pattern is in line with the observed effect on slug preference in local leaves. In systemic leaves, maximal resistance was already achieved after 24 h feeding. This result suggests that young systemic leaves more rapidly attain effective levels of defense metabolites, such as glycoalkaloids (see above), than older local leaves, possibly via rapid translocation of glycoalkaloids and other defenses (van Dam et al., 1995). This is in agreement with the optimal defense hypothesis, in which younger leaves are more valuable to the plant, have higher constitutive defense levels, and are more inducible than older leaves (Meldau et al., 2012).

Finally, our experimental set-up allowed us to reveal geographic variation among populations for slug-induced responses. The transcriptome profiles of *S. dulcamara* plants clearly showed a core set of GFS induced responses, but German and Dutch populations separated on the second axis of the PCA (Figure 4A). Similar patterns were found in a panel of eight *A. thaliana* accessions subjected to flooding (van Veen et al., 2016). Despite the genotypic differences, they identified a core set of flooding specific transcripts. In order to separate general slug-induced responses from genotypic or population specific responses, a wider set of *S. dulcamara* accessions should be screened.

In conclusion, our results illustrate that induced responses and resistance to GFS feeding in *S. dulcamara* are regulated by similar pathways and metabolites as those involved in insect induced responses (Lortzing et al., 2017; Geuss et al., 2018; Nguyen et al., 2018). Yet, plants did not downregulate the transcription of photosynthesis-related genes upon slug feeding, which is in contrast to what commonly happens after insect feeding. This illustrates that gastropods and insects may induce specific responses, despite of similarities in the regulatory pathways they induce. Highly standardized experiments comparing the response of *S. dulcamara* to various gastropod and insect herbivores are required to assess in how far induction patterns are specific to different classes of herbivores. Besides, we show that relaxation plays a role in the dynamics of GFS-induced defenses, and that sustained feeding can boost the locally induced response. The temporal and spatial dynamics of GFS-induced responses, in particular with respect to those related to primary and secondary metabolism, illustrate that *S. dulcamara* plants possibly balance resource investment after induction. Together with the rapidly induced local and systemic resistance, we may conclude that *S. dulcamara* shows a functional response to GFS, which is well-balanced with the incidence of feeding damage. Depending on the turnover rate of defense compounds in the absence of feeding, this induced response may have consequences for other herbivores in the community (Viswanathan et al., 2005). Further elucidation of the specific interactions between gastropods and plants may therefore provide novel insights to the general regulation and diversity of plant–herbivore interactions.

## Data Availability Statement

The datasets presented in this study can be found in online repositories. The names of the repository/repositories and accession number(s) can be found in the article/Supplementary Material.

## Author Contributions

OC, ND, AS, HH, and JP conceived and designed the experiment and took the lead in writing the manuscript. All other co-authors contributed to the manuscript by giving comprehensive feedback. OC performed the experiments and analyzed the data. TL performed and assisted in transcriptomic and phytohormone analyses. AW and YP performed the metabolomics analyses and supported the metabolomic data analyses.

## Conflict of Interest

The authors declare that the research was conducted in the absence of any commercial or financial relationships that could be construed as a potential conflict of interest.

## Footnotes

1 www.agilent.com

2 www.ncbi.nlm.nih.gov/tools/primer-blast/

3 solgenomics.net/

## References

[B2] Agrawal A. A. (1998). Induced responses to herbivory and increased plant performance. *Science* 279 1201–1202. 10.1126/science.279.5354.1201 9469809

[B3] Agrawal A. A. (2000). Specificity of induced resistance in wild radish: causes and consequences for two specialist and two generalist caterpillars. *Oikos* 89 493–500. 10.1034/j.1600-0706.2000.890308.x

[B4] Alexa A. Rahnenfuhrer J. (2010). *topGO: Enrichment Analysis for Gene Ontology. R package, version 2.22.0.*

[B5] Alexa A. Rahnenfuhrer J. Lengauer T. (2006). Improved scoring of functional groups from gene expression data by decorrelating GO graph structure. *Bioinformatics* 22 1600–1607. 10.1093/bioinformatics/btl140 16606683

[B6] Backmann P. Grimm V. Jetschke G. Lin Y. Vos M. Baldwin I. T. (2019). Delayed chemical defense: timely expulsion of herbivores can reduce competition with neighboring plants. *Am. Nat.* 193 125–139. 10.1086/700577 30624112

[B7] Baldwin I. T. (1996). Allometric limits to the induced accumulation of nicotine in native tobacco. *Plant Spec. Biol.* 11 107–114. 10.1111/j.1442-1984.1996.tb00113.x

[B8] Basu S. Varsani S. Louis J. (2018). Altering plant defenses: herbivore-associated molecular patterns and effector arsenal of chewing herbivores. *Mol. Plant Microbe Interact.* 31 13–21. 10.1094/mpmi-07-17-0183-fi 28840787

[B9] Beckers G. J. M. Spoel S. H. (2006). Fine-tuning plant defence signalling: salicylate versus jasmonate. *Plant Biol.* 8 1–10. 10.1055/s-2005-872705 16435264

[B10] Bilgin D. D. Zavala J. A. Zhu J. Clough S. J. Ort D. R. DeLucia E. H. (2010). Biotic stress globally downregulates photosynthesis genes. *Plant Cell Environ.* 33 1597–1613. 10.1111/j.1365-3040.2010.02167.x 20444224

[B11] Bode R. F. Halitschke R. Kessler A. (2013). Herbivore damage-induced production and specific anti-digestive function of serine and cysteine protease inhibitors in tall goldenrod, *Solidago altissima* L. (Asteraceae). *Planta* 237 1287–1296. 10.1007/s00425-013-1845-9 23371287

[B12] Boege K. Marquis R. J. (2005). Facing herbivory as you grow up: the ontogeny of resistance in plants. *Trends Ecol. Evol.* 20 441–448. 10.1016/j.tree.2005.05.001 16701415

[B13] Bonaventure G. VanDoorn A. Baldwin I. T. (2011). Herbivore-associated elicitors: FAC signaling and metabolism. *Trends Plant Sci.* 16 294–299. 10.1016/j.tplants.2011.01.006 21354852

[B14] Bradford M. M. (1976). A rapid and sensitive method for the quantitation of microgram quantities of protein utilizing the principle of protein-dye binding. *Anal. Biochem.* 72 248–254. 10.1016/0003-2697(76)90527-3 942051

[B15] Calf O. W. (2019). *B’sweet or B’sour—An Ecologica, Metabolomic and Molecular Analysis of Slug Resistance in Solanum dulcamara.* Ph.D. thesis, Radboud University, Nijmegen NL.

[B16] Calf O. W. Huber H. Peters J. L. Weinhold A. Poeschl Y. van Dam N. M. (2019). Gastropods and insects prefer different *Solanum dulcamara* chemotypes. *J. Chem. Ecol.* 45 146–161. 10.1007/s10886-018-0979-4 29961916 PMC6469604

[B17] Calf O. W. Huber H. Peters J. L. Weinhold A. van Dam N. M. (2018). Glycoalkaloid composition explains variation in slug resistance in *Solanum dulcamara*. *Oecologia* 2 495–506. 10.1007/s00442-018-4064-z PMC5997107 29383505

[B18] Calf O. W. van Dam N. M. (2012). Bittersweet bugs: the Dutch insect community on the nightshade *Solanum dulcamara*. *Entomolog. Ber.* 72 193–198.

[B19] Chong J. Soufan O. Li C. Caraus I. Li S. Bourque G. (2018). MetaboAnalyst 4.0: towards more transparent and integrative metabolomics analysis. *Nucleic Acids Res.* 46 W486–W494. 10.1093/nar/gky310 29762782 PMC6030889

[B20] Chung S. H. Felton G. W. (2011). Specificity of induced resistance in tomato against specialist lepidopteran and coleopteran species. *J. Chem. Ecol.* 37 378–386. 10.1007/s10886-011-9937-0 21455676

[B21] Chung S. H. Rosa C. Hoover K. Luthe D. S. Felton G. W. (2013). Colorado potato beetle manipulates plant defenses in local and systemic leaves. *Plant Signal. Behav.* 8:e27592 10.4161/psb.27592 PMC4091235 24390091

[B22] D’Agostino N. Golas T. van de Geest H. Bombarely A. Dawood T. Zethof J. (2013). Genomic analysis of the native European *Solanum* species, *S. dulcamara*. *BMC Genomics* 14:356 10.1186/1471-2164-14-356 PMC3680029 23713999

[B23] Danner H. Desurmont G. A. Cristescu S. M. van Dam N. M. (2017). Herbivore-induced plant volatiles accurately predict history of coexistence, diet breadth, and feeding mode of herbivores. *New Phytol.* 220 726–738. 10.1111/nph.14428 28134434

[B24] de Mendiburu F. (2017). *agricolae: Statistical Procedures for Agricultural Research. R package, version 1.2–8.*

[B25] de Vos M. van Oosten V. R. van Poecke R. M. P. van Pelt J. A. Pozo M. J. Müller M. J. (2005). Signal signature and transcriptome changes of *Arabidopsis* during pathogen and insect attack. *Mol. Plant Microbe Interact.* 18 923–937. 10.1094/mpmi-18-0923 16167763

[B26] Desurmont G. A. Zemanova M. A. Turlings T. C. J. (2016). The gastropod menace: slugs on Brassica plants affect caterpillar survival through consumption and interference with parasitoid attraction. *J. Chem. Ecol.* 42 183–192. 10.1007/s10886-016-0682-2 27002323

[B27] Erb M. Meldau S. Howe G. A. (2012). Role of phytohormones in insect-specific plant reactions. *Trends Plant Sci.* 17 250–259. 10.1016/j.tplants.2012.01.003 22305233 PMC3346861

[B28] Falk K. L. Kästner J. Bodenhausen N. Schramm K. Paetz C. Vassão D. G. (2014). The role of glucosinolates and the jasmonic acid pathway in resistance of *Arabidopsis thaliana* against molluscan herbivores. *Mol. Ecol.* 23 1188–1203. 10.1111/mec.12610 24313595 PMC5147714

[B29] Fox J. Weisberg S. (2011). *An R Companion to Applied Regression*, 2nd Edn Thousand Oaks, CA: SAGE Publications Inc, 472.

[B30] Gaquerel E. Gulati J. Baldwin I. T. (2014). Revealing insect herbivory-induced phenolamide metabolism: from single genes to metabolic network plasticity analysis. *Plant J.* 79 679–692. 10.1111/tpj.12503 24617849 PMC5140026

[B31] Geuss D. Lortzing T. Schwachtje J. Kopka J. Steppuhn A. (2018). Oviposition by *Spodoptera exigua* on *Solanum dulcamara* alters the plant’s response to herbivory and impairs larval performance. *Int. J. Mol. Sci.* 19:4008 10.3390/ijms19124008 PMC6321313 30545097

[B32] Geuss D. Stelzer S. Lortzing T. Steppuhn A. (2017). *Solanum dulcamara*’s response to eggs of an insect herbivore comprises ovicidal hydrogen peroxide production. *Plant Cell Environ.* 40 2663–2677. 10.1111/pce.13015 28667817

[B33] Glawe G. A. Zavala J. A. Kessler A. van Dam N. M. Baldwin I. T. (2003). Ecological costs and benefits correlated with trypsin protease inhibitor production in *Nicotiana attenuata*. *Ecology* 84 79–90. 10.1890/0012-9658(2003)084[0079:ecabcw]2.0.co;2

[B34] Gould K. S. (2004). Nature’s Swiss army knife: the diverse protective roles of anthocyanins in leaves. *J. Biomed. Biotechnol.* 5 314–320. 10.1155/s1110724304406147 PMC1082902 15577195

[B35] Heil M. Ibarra-Laclette E. Adame-Álvarez R. M. Martínez O. Ramirez-Chávez E. Molina-Torres J. (2012). How plants sense wounds: damaged-self recognition is based on plant-derived elicitors and induces octadecanoid signaling. *PLoS One* 7:e30537 10.1371/journal.pone.0030537 PMC3276496 22347382

[B36] Howe G. A. Jander G. (2008). Plant immunity to insect herbivores. *Annu. Rev. Plant Biol.* 59 41–66. 10.1146/annurev.arplant.59.032607.092825 18031220

[B37] Karban R. (2011). The ecology and evolution of induced resistance against herbivores. *Funct. Ecol.* 25 339–347. 10.1111/j.1365-2435.2010.01789.x

[B38] Karban R. Baldwin I. T. (1997). *Induced Responses to Herbivory.* Chicago, IL: University of Chicago Press, 330.

[B39] Kästner J. von Knorre D. Himanshu H. Erb M. Baldwin I. T. Meldau S. (2014). Salicylic acid, a plant defense hormone, is specifically secreted by a molluscan herbivore. *PLoS One* 9:e86500 10.1371/journal.pone.0086500 PMC3899270 24466122

[B40] Khan M. B. Harborne J. B. (1990). Induced alkaloid defence in *Atropa acuminata* in response to mechanical and herbivore leaf damage. *Chemoecology* 1 77–80. 10.1007/bf01325232

[B41] Korell L. Stein C. Hensen I. Bruelheide H. Suding K. N. Auge H. (2016). Stronger effect of gastropods than rodents on seedling establishment, irrespective of exotic or native plant species origin. *Oikos* 125 1467–1477. 10.1111/oik.02696

[B42] Lawrence S. D. Novak N. G. Ju C. J. T. Cooke J. E. K. (2008). Potato, *Solanum tuberosum*, defense against Colorado potato beetle, *Leptinotarsa decemlineata* (Say): microarray gene expression profiling of potato by Colorado potato beetle regurgitant treatment of wounded leaves. *J. Chem. Ecol.* 34 1013–1025. 10.1007/s10886-008-9507-2 18581175

[B43] Leon J. Rojo E. Sanchez-Serrano J. J. (2001). Wound signalling in plants. *J. Exp. Bot.* 52 1–9. 10.1093/jexbot/52.354.1 11181708

[B44] Lorenzo O. Solano R. (2005). Molecular players regulating the jasmonate signalling network. *Curr. Opin. Plant Biol.* 8 532–540. 10.1016/j.pbi.2005.07.003 16039901

[B45] Lortzing T. Firtzlaff V. Nguyen D. Rieu I. Stelzer S. Schad M. (2017). Transcriptomic responses of *Solanum dulcamara* to natural and simulated herbivory. *Mol. Ecol. Resour.* 17 e196–e211. 10.1111/1755-0998.12687 28449359

[B46] Maffei M. E. Mithöfer A. Boland W. (2007). Insects feeding on plants: rapid signals and responses preceding the induction of phytochemical release. *Phytochemistry* 68 2946–2959. 10.1016/j.phytochem.2007.07.016 17825328

[B47] Mason C. J. Villari C. Keefover-Ring K. Jagemann S. Zhu J. Bonello P. (2017). Spatial and temporal components of induced plant responses in the context of herbivore life history and impact on host. *Funct. Ecol.* 31 2034–2050. 10.1111/1365-2435.12911

[B48] Mathur V. Ganta S. Raaijmakers C. E. Reddy A. S. Vet L. E. M. van Dam N. M. (2011). Temporal dynamics of herbivore-induced responses in *Brassica juncea* and their effect on generalist and specialist herbivores. *Entomol. Exp. Appl.* 139 215–225. 10.1111/j.1570-7458.2011.01122.x

[B49] Meldau S. Erb M. Baldwin I. T. (2012). Defence on demand: mechanisms behind optimal defence patterns. *Ann. Bot.* 110 1503–1514. 10.1093/aob/mcs212 23022676 PMC3503495

[B50] Meldau S. Kästner J. von Knorre D. Baldwin I. T. (2014). Salicylic acid-dependent gene expression is activated by locomotion mucus of different molluscan herbivores. *Commun. Integr. Biol.* 7:e28728 10.4161/cib.28728 PMC4203486 25346792

[B51] Mellway R. D. Tran L. T. Prouse M. B. Campbell M. M. Constabel C. P. (2009). The wound-, pathogen-, and Ultraviolet B-responsive MYB134 gene encodes an R2R3 MYB transcription factor that regulates proanthocyanidin synthesis in poplar. *Plant Physiol.* 150 924–941. 10.1104/pp.109.139071 19395405 PMC2689947

[B52] Mithöfer A. Boland W. (2008). Recognition of herbivory-associated molecular patterns. *Plant Physiol.* 146 825–831. 10.1104/pp.107.113118 18316636 PMC2259064

[B53] Nakata M. Ohme-Takagi M. (2014). Quantification of anthocyanin content. *Bio Protoc.* 4:e1098 10.21769/BioProtoc.1098

[B54] Nguyen D. D’Agostino N. Tytgat T. O. G. Sun P. L. Lortzing T. Visser E. J. W. (2016a). Drought and flooding have distinct effects on herbivore-induced responses and resistance in *Solanum dulcamara*. *Plant Cell Environ.* 39 1485–1499. 10.1111/pce.12708 26759219

[B55] Nguyen D. Rieu I. Mariani C. van Dam N. M. (2016b). How plants handle multiple stresses: hormonal interactions underlying responses to abiotic stress and insect herbivory. *Plant Mol. Biol.* 91 727–740. 10.1007/s11103-016-0481-8 27095445 PMC4932144

[B56] Nguyen D. Poeschl Y. Lortzing T. Hoogveld R. Gogol-Döring A. Cristescu S. M. (2018). Interactive responses of *Solanum dulcamara* to drought and insect feeding are herbivore species-specific. *Int. J. Mol. Sci.* 19:3845 10.3390/ijms19123845 PMC6321310 30513878

[B57] Núñez-Farfán J. Fornoni J. Valverde P. L. (2007). The evolution of resistance and tolerance to herbivores. *Annu. Rev. Ecol. Evol. Sci.* 38 541–566. 10.1146/annurev.ecolsys.38.091206.095822

[B58] Orians C. M. Thorn A. Gomez S. (2011). Herbivore-induced resource sequestration in plants: why bother? *Oecologia* 167 1–9. 10.1007/s00442-011-1968-2 21431939

[B59] Orrock J. L. (2013). Exposure of unwounded plants to chemical cues associated with herbivores leads to exposure-dependent changes in subsequent herbivore attack. *PLoS One* 8:e79900 10.1371/journal.pone.0079900 PMC3835831 24278210

[B60] Peters K. Worrich A. Weinhold A. Alka O. Balcke G. Birkemeyer C. (2018). Current challenges in plant eco-metabolomics. *Int. J. Mol. Sci.* 19:1385 10.3390/ijms19051385 PMC5983679 29734799

[B61] Pohlert T. (2014). *The Pairwise Multiple Comparison of Mean Ranks Package (PMCMR). R package, version 2016-01–06.*

[B62] R Core Team, (2016). *R: A Language and Environment for Statistical Computing.* Vienna: R Foundation for Statistical Computing.

[B63] Raj S. N. Sarosh B. R. Shetty H. S. (2006). Induction and accumulation of polyphenol oxidase activities as implicated in development of resistance against pearl millet downy mildew disease. *Funct. Plant Biol.* 33 563–571. 10.1071/FP06003 32689264

[B64] Schwachtje J. Baldwin I. T. (2008). Why does herbivore attack reconfigure primary metabolism? *Plant Physiol.* 146 845–851. 10.1104/pp.107.112490 18316639 PMC2259057

[B65] Schwachtje J. Minchin P. E. H. Jahnke S. van Dongen J. T. Schittko U. Baldwin I. T. (2006). SNF1-related kinases allow plants to tolerate herbivory by allocating carbon to roots. *Proc. Natl. Acad. Sci. U.S.A.* 103 12935–12940. 10.1073/pnas.0602316103 16912118 PMC1568949

[B66] Schweiger R. Heise A. M. Persicke M. Müller C. (2014). Interactions between the jasmonic and salicylic acid pathway modulate the plant metabolome and affect herbivores of different feeding types. *Plant Cell Environ.* 37 1574–1585. 10.1111/pce.12257 24372400

[B67] Steinbrenner A. D. Gomez S. Osorio S. Fernie A. R. Orians C. M. (2011). Herbivore-induced changes in tomato (*Solanum lycopersicum*) primary metabolism: a whole plant perspective. *J. Chem. Ecol.* 37 1294–1303. 10.1007/s10886-011-0042-1 22161151

[B68] Stout M. J. (2013). Reevaluating the conceptual framework for applied research on host-plant resistance. *Insect Sci.* 20 263–272. 10.1111/1744-7917.12011 23955879

[B69] Stowe K. A. Marquis R. J. Hochwender C. G. Simms E. L. (2000). The evolutionary ecology of tolerance to consumer damage. *Annu. Rev. Ecol. Syst.* 31 565–595. 10.1146/annurev.ecolsys.31.1.565

[B70] Strauss S. Y. Agrawal A. A. (1999). The ecology and evolution of plant tolerance to herbivory. *Trends Ecol. Evol.* 14 179–185. 10.1016/s0169-5347(98)01576-6 10322530

[B71] Strauss S. Y. Stanton M. L. Emery N. C. Bradley C. A. Carleton A. Dittrich-Reed D. R. (2009). Cryptic seedling herbivory by nocturnal introduced generalists impacts survival, performance of native and exotic plants. *Ecology* 90 419–429. 10.1890/07-1533.1 19323226

[B72] Tang J. Y. Zielinski R. E. Zangerl A. R. Crofts A. R. Berenbaum M. R. DeLucia E. H. (2006). The differential effects of herbivory by first and fourth instars of *Trichoplusia ni* (Lepidoptera: Noctuidae) on photosynthesis in *Arabidopsis thaliana*. *J. Exp. Bot.* 57 527–536. 10.1093/jxb/erj032 16377737

[B73] Thaler J. S. Humphrey P. T. Whiteman N. K. (2012). Evolution of jasmonate and salicylate signal crosstalk. *Trends Plant Sci.* 17 260–270. 10.1016/j.tplants.2012.02.010 22498450

[B74] Thaler J. S. Stout M. J. Karban R. Duffey S. S. (1996). Exogenous jasmonates simulate insect wounding in tomato plants (*Lycopersicon esculentum*) in the laboratory and field. *J. Chem. Ecol.* 22 1767–1781. 10.1007/bf02028503 24227107

[B75] Underwood N. (2012). When herbivores come back: effects of repeated damage on induced resistance. *Funct. Ecol.* 26 1441–1449. 10.1111/j.1365-2435.2012.02055.x

[B76] van Dam N. M. Raaijmakers C. E. (2006). Local and systemic induced responses to cabbage root fly larvae (*Delia radicum*) in *Brassica nigra* and *B. oleracea*. *Chemoecology* 16 17–24. 10.1007/s00049-005-0323-7

[B77] van Dam N. M. Witte L. Theuring C. Hartmann T. (1995). Distribution, biosynthesis and turnover of pyrrolizidine alkaloids in *Cynoglossum officinale*. *Phytochemistry* 39 287–292. 10.1016/0031-9422(94)00944-o

[B78] van der Meijden E. Wijn M. Verkaar H. J. (1988). Defense and regrowth, alternative plant strategies in the struggle against herbivores. *Oikos* 51 355–363.

[B79] van Veen H. Vashisht D. Akman M. Girke T. Mustroph A. Reinen E. (2016). Transcriptomes of eight *Arabidopsis thaliana* accessions reveal core conserved, genotype- and organ-specific responses to flooding stress. *Plant Physiol.* 172 668–689. 27208254 10.1104/pp.16.00472 PMC5047075

[B80] van Zandt P. A. Agrawal A. A. (2004). Specificity of induced plant responses to specialist herbivores of the common milkweed *Asclepias syriaca*. *Oikos* 104 401–409. 10.1111/j.0030-1299.2004.12964.x

[B81] Viswanathan D. V. Narwani A. J. T. Thaler J. S. (2005). Specificity in induced plant responses shapes patterns of herbivore occurrence on *Solanum dulcamara*. *Ecology* 86 886–896. 10.1890/04-0313

[B82] Viswanathan D. V. Thaler J. S. (2004). Plant vascular architecture and within-plant spatial patterns in resource quality following herbivory. *J. Chem. Ecol.* 30 531–543. 10.1023/b:joec.0000018627.26420.e0 15139306

[B83] Voelckel C. Baldwin I. T. (2004). Generalist and specialist lepidopteran larvae elicit different transcriptional responses in *Nicotiana attenuata*, which correlate with larval FAC profiles. *Ecol. Lett.* 7 770–775. 10.1111/j.1461-0248.2004.00633.x

[B84] Walker A. J. Glen D. M. Shewry P. R. (1998). Purification and characterization of a digestive cysteine proteinase from the field slug (*Deroceras reticulatum*): a potential target for slug control. *J. Agric. Food Chem.* 46 2873–2881.

[B85] Walker A. J. Urwin P. E. Atkinson H. J. Brain P. Glen D. M. Shewry P. R. (1999). Transgenic Arabidopsis leaf tissue expressing a modified oryzacystatin shows resistance to the field slug *Deroceras reticulatum* (Müller). *Transgenic Res.* 8 95–103. 10481309 10.1023/a:1008814317199

[B86] Walling L. L. (2000). The myriad plant responses to herbivores. *J. Plant Growth Regul.* 19 195–216. 11038228 10.1007/s003440000026

[B87] Westerhuis J. A. Hoefsloot H. C. J. Smit S. Vis D. J. Smilde A. K. van Velzen E. J. J. (2008). Assessment of PLSDA cross validation. *Metabolomics* 4 81–89.

[B88] Wiklund S. Johansson E. Sjostrom L. Mellerowicz E. J. Edlund U. Shockcor J. P. (2008). Visualization of GC/TOF-MS-based metabolomics data for identification of biochemically interesting compounds using OPLS class models. *Anal. Chem.* 80 115–122. 18027910 10.1021/ac0713510

[B89] Wingler A. Roitsch T. (2008). Metabolic regulation of leaf senescence: interactions of sugar signalling with biotic and abiotic stress responses. *Plant Biol.* 10 50–62. 18721311 10.1111/j.1438-8677.2008.00086.x

[B90] Wintermans J. F. Demots A. (1965). Spectrophotometric characteristics of chlorophylls a and b and their pheophytins in ethanol. *Biochim. Biophys. Acta* 109 448–453. 5867546 10.1016/0926-6585(65)90170-6

[B91] Wittstock U. Gershenzon J. (2002). Constitutive plant toxins and their role in defense against herbivores and pathogens. *Curr. Opin. Plant Biol.* 5 300–307. 12179963 10.1016/s1369-5266(02)00264-9

[B92] Worley B. Powers R. (2013). Multivariate analysis in metabolomics. *Curr. Metab.* 1 92–107. 10.2174/2213235X11301010092 PMC4465187 26078916

[B93] Zangerl A. R. Hamilton J. G. Miller T. J. Crofts A. R. Oxborough K. Berenbaum M. R. (2002). Impact of folivory on photosynthesis is greater than the sum of its holes. *Proc. Natl. Acad. Sci. U.S.A.* 99 1088–1091. 11792866 10.1073/pnas.022647099 PMC117434

[B94] Zhang Q. Peters J. L. Visser E. J. W. de Kroon H. Huber H. (2016). Hydrologically contrasting environments induce genetic but not phenotypic differentiation in *Solanum dulcamara*. *J. Ecol.* 104 1649–1661.

[B95] Zhou S. Lou Y. R. Tzin V. Jander G. (2015). Alteration of plant primary metabolism in response to insect herbivory. *Plant Physiol.* 169 1488–1498. 26378101 10.1104/pp.15.01405 PMC4634104

[B96] Zhu X. Y. Chen J. Y. Qiu K. Kuai B. K. (2017). Phytohormone and light regulation of chlorophyll degradation. *Front. Plant Sci.* 8:1911 10.3389/fpls.2017.01911 PMC5681529 29163624

[B97] Zhu-Salzman K. Zeng R. (2015). Insect response to plant defensive protease inhibitors. *Annu. Rev. Entomol.* 60 233–252. 25341101 10.1146/annurev-ento-010814-020816

