# Supplementary material for: Slug Feeding Triggers Dynamic Metabolomic and Transcriptomic Responses Leading to Induced Resistance in Solanum dulcamara
Source: Front Plant Sci. 2020 Jun 18;11:803. doi: 10.3389/fpls.2020.00803 (PMC7314995; doi:10.3389/fpls.2020.00803)
Supplement: Supplementary file 1 [file Data_Sheet_1.docx]

**Document S1** Protocols for untargeted metabolic profiling, phytohormone quantification and RT-qPCR validation of microarray

*Metabolic profiling*

*Extraction*

Fresh leaves were ground in liquid nitrogen. About 100 mg of each sample was double extracted with respectively 1.0 and 0.9 ml MeOH:Acetate (50/50, v/v%) buffer (pH 4.8) in 2 ml reaction tubes holding two glass beads (5 mm Ø) by shaking in a TissueLyser (Qiagen, Venlo, the Netherlands) at 50 Hz for 5 minutes followed by centrifugation at 14.000 rpm at 4 °C. Clear supernatants were combined and stored at -20 °C until further processing.

*LC-qToF-MS analyses*

Two sets of diluted crude extracts (1:5 and 1:50) were analysed with an UltiMate™ 3000 Standard Ultra-High-Pressure Liquid Chromatography system (UHPLC, Thermo Fisher Scientific).

The 1:5 dilutions were analysed using an Acclaim® Rapid Separation Liquid Chromatography (RSLC) 120 column (100 × 2.1 mm, particle size 2.2 μm, Thermo Fisher Scientific) and the following gradient at a flow rate of 0.4 mL / min^-1^: 0–2 min isocratic 95% A (water / formic acid 99.95/0.05 (v/v%)), 5% B (acetonitrile / formic acid 99.95/0.05 (v/v%)); 2–7 min, linear from 5% to 30% B; 7–12 min, linear from 30% to 35% B; 12-15 min, linear from 35% to 95% B; 15–17 min, isocratic 95% B; 17–19 min, linear from 95% to 5% B; 19–23 min, isocratic 5% B. Metabolites were detected with a maXis impact™ quadrupole time-of-flight mass spectrometer (qToF-MS, Bruker Daltonics) applying the following conditions in positive mode: scan range 50–1000 *m/z*; acquisition rate 3 Hz; end plate offset 500 V; capillary voltage 3500 V; nebulizer pressure 2 bar, dry gas 10 L min^-1^, dry temperature 220 °C. Mass calibration was performed using sodium formate clusters (10 mM solution of NaOH in 50/50 (v/v%) isopropanol water containing 0.2% formic acid.

To achieve a better chromatographic separation, the 1:50 diluted extracts were analysed using a Acclaim® Rapid Separation Liquid Chromatography (RSLC) 120 column (150 × 2.1 mm, particle size 2.2 μm, Thermo Fisher Scientific) and the following gradient at a flow rate of 0.4 mL/min^-1^: 0–2 min isocratic 95% A (water/formic acid 99.95/0.05 (v/v%)), 5% B (acetonitrile/formic acid 99.95/0.05 (v/v%)); 2–15 min, linear from 5% to 40% B; 15–20 min, linear from 40% to 95% B; 20–22 min, isocratic 95% B; 22–25 min, linear from 95% to 5% B; 25–30 min, isocratic 5% B. Metabolites were detected with a maXis impact™ quadrupole time-of-flight mass spectrometer (qToF-MS, Bruker Daltonics) applying the following conditions in positive mode: scan range 50–1400 *m/z*; acquisition rate 3 Hz; end plate offset 500 V; capillary voltage 3500 V; nebulizer pressure 2 bar, dry gas 10 L min^-1^, dry temperature 220 °C. Mass calibration was performed using sodium formate clusters (10 mM solution of NaOH in 50/50 (v/v%) isopropanol water containing 0.2% formic acid.

*Data processing*

The LC-qToF-MS raw data were recalibrated and then converted to the mzXML format by using the CompassXport utility of the DataAnalysis software (Bruker Daltonik GmbH, Bremen, Germany). Mass spectra of the dataset obtained from analysing the 1:5 diluted samples were processed using XCMS and CAMERA packages in R (Smith *et al*., 2006; Kuhl *et al*., 2012). The datasets from local and systemic leaves were processed separately, because of the large fraction of position specific metabolites. Automated peak picking and alignment were done within a retention time between 50–480 seconds, signal:noise ratio ≥ 50, maximum deviation of 5 ppm, 3 seconds retention time window, mass/charge window of 0.005 and minimum occurrence in four out of six samples in at least one treatment. Resulting features were grouped to belong to the same metabolite after symmetric retention time correction, within a retention time window of maximum 5 seconds and minimum mutual correlation of 0.8. Feature intensities were multiplied by their dilution factor (5) and samples normalised by the fresh weight of the leaf material used for extraction. Missing values were replaced by the treatment mean value only when one of six replicates was missing or else by a random value between 1 and half of the minimum value in the complete dataset. Feature groups, potentially representing single metabolites, were reduced to one feature to represent the respective metabolite in later analysis. This was done by applying the in-house maximum heuristic approach, which selects the feature with the highest intensity in the majority of the samples.

*Relative quantification of highly abundant metabolites*

Peak intensities of four putatively identified glycoalkaloids (α-solamarine, β-solamarine, solasonine and solamargine; all with retention times greater than 480 sec) exceeded the saturation intensity threshold and were therefore manually quantified from the 1:50 diluted samples. Values corresponding to these metabolites in the automatically processed dataset were replaced by the manually quantified intensities after dilution correction.

*Putative identification of metabolites of interest*

Putative identification was based on (Calf *et al*. 2019). Tandem mass spectrometry (MS^2^) spectra were acquired by injection of samples that contained the highest amount of the metabolites of interest. The separation was achieved by using the same chromatographic conditions as described above. MS^2^ spectra were collected by using the automated MSMS function of the Bruker oToF Control software. Spectra were evaluated for metabolites of interest with particular emphasis on fragmentation of the parental metabolite.

*Targeted analyses of induced responses*

The levels of total proteins, polyphenol oxidase (PPO) activity, trypsin protease inhibitor (TPI) activity, chlorophyll and anthocyanin in local and systemic leaves were quantified spectrophotometrically. Three technical replicates were analysed in microplate assays on a SpectraMax® 190 absorbance plate reader (Molecular Devices, San Jose, USA) using SoftMax® Pro software.

The levels of total proteins, PPO activity and TPI were quantified from the same protein extract. This was obtained from 90–110 mg ground fresh leaf material (weighed to the nearest decimal). Extraction was performed in 2 ml reaction tubes holding two glass beads (5 mm Ø) with 0.8 ml 0.1 M KPO_4_ buffer (pH 7.3) containing 5% (w/v) PVPP (Sigma-Aldrich, Zwijndrecht, the Netherlands, P6755) and 0.83% (v/v) Triton™ X-100 (Sigma-Aldrich, T8787). Reaction tubes were shaken twice at 50 Hz using a TissueLyser (Qiagen, Venlo, the Netherlands) for 30 seconds with a 60 second interval, followed by centrifugation for five minutes at 14.000 rpm by 4 °C. The clear supernatant was stored at -20 °C until further processing. Total protein content (mg g^-1^ FW) was determined relative to a 7 point concentration series (10–500 mg ml^-1^) of albumin (735078, Boehringer-Mannheim, Mannheim, Germany) at 595 nm using the Bradford method (Bradford 1976). The activity of PPO was quantified by the change in absorbance at 435 nm (ΔOD_435_ min^-1^ mg^-1^ protein) using 2.92 mM caffeic acid (Sigma-Aldrich, C0625) as substrate following a method derived from Thaler *et al.* (1996). We quantified TPI activity (μg mg^-1^ protein) relative to a 6 point concentration series (10–100 µg ml^-1^) of soybean TPI (Sigma-Aldrich, T9003) following the method derived from (Bode *et al.* 2013). We used 0.2 mg ml^-1^ bovine trypsin protease (Sigma-Aldrich, T8003) as reference enzyme and N-benzoyl-DL-arginine-ß-naphtylamide hydrochloride (BANA, Sigma-Aldrich, B4750) as substrate. TPI activity was calculated from the difference in absorbance at 550 nm.

Total chlorophyll levels were quantified using to the method described by Wintermans & Demots (1965). A leaf extract was obtained from 30–40 mg ground fresh leaf material (weighed to the nearest decimal). Extraction was performed in 2 ml reaction tubes holding two glass beads (5 mm Ø) with 1.8 ml absolute EtOH (Emsure®, Sigma-Aldrich). Reaction tubes were shaken three times at 50 Hz using a TissueLyser (Qiagen) for 30 seconds with a 60 second interval, followed by centrifugation for five minutes at 14.000 rpm by 4 °C. We measured the absorbance of the clear supernatant at 654 nm and calculated the total chlorophyll levels (mg g^-1^ FW) with the reported absorbance coefficient (39.8 g^-1^ cm^-1^)using the following formula:

$$\left[ \mathrm{Ext}_{654}-\mathrm{EtOH}_{654} \right]*\frac{1.8}{Volume used \left( \mathrm{ml} \right)}*\frac{1}{39.8}*\frac{1}{FW (g)}$$

Anthocyanin levels were quantified following the protocol by Nakata & Ohme-Takagi (2014). A leaf extract was obtained from 75–90 mg ground fresh leaf material (weighed to the nearest decimal). Extraction was performed in 2 ml reaction tubes holding two glass beads (5 mm Ø) with 0.6 ml extraction buffer of 45% MeOH and 5% AcOH in water (pH 2.68). Reaction tubes were shaken three times at 50 Hz using a TissueLyser (Qiagen) for 30 seconds with a 60 second interval, followed by centrifugation for two minutes at 14.000 rpm by 4 °C. We measured the absorbance of the clear supernatant at 530 and 657 nm and calculated the relative anthocyanin levels (OD_530_ g^-1^ FW) using the following formula:

$$\left[ \mathrm{Ext}_{530}-\mathrm{Buffer}_{530} \right]-(0.25*\left[ \mathrm{Ext}_{657}-\mathrm{Buffer}_{657} \right]*\frac{0.6}{Volume used \left( \mathrm{ml} \right)}*\frac{1}{FW (g)}$$

*Mass spectrometry for phytohormone measurements*

Mass spetrometry for phytohormone measurements: A Synapt G2-S HDMS (Waters®, Milford, Massachusetts, USA) was used for phytohormone separation, detection, and quantification. A sample of 7 µl was injected into the UPLC system AQUITY™, Waters, Milford Massachusetts USA) and separated on a C18 column (Acquity UPLC Waters, BEH-C18, Ø 2.1 mm x 50 mm, particle size 1.7 µm). In a gradient separation, water and methanol (each with 0.1% formic acid (v/v) were used as eluents A and B with constant flow of 250 µl/min at 30°C (eluent B: 0 min: 30%; 1 min: 30%; 4.5 min: 90%; 8 min: 90%; 9 min: 30%; 3 min equilibration time between the runs). The separated compounds were negatively ionized by electrospraying (ESI) at the following conditions: capillary voltage 2.5 kV, nebulizer 6 bar, desolvation gas flow rate 500 l/hour, 80°C source temperature and 150°C desolvation temperature, N_2_ as desolvation gas. Compounds were detected by tandem mass spectrometry scanning the full mass spectrum of compounds between 50 and 600 m/z. Compounds were fragmented in the ion mobility cell with 2 V and 4 V collision energy on the transfer- and trap lense clusters respectively. The phytohormonal compounds were annotated according to their parent [M-H]- ion and a diagnostic daughter ion as well as according to co-elution with their deuterated derivatives (see supplemental table S1 for details).

*RT-qPCR for microarray validation*

New total RNA isolates were made from pools of ground leaf tissue, each including the four plants of a single plant population in each treatment, as hybridized on the microarray. Extraction and purification were performed using the RNeasy® Plant Mini kit (Qiagen) and DNAse I, RNAse-free (Fermentas) following the manufacturer’s instructions. Then, cDNA was synthesised from ~1000 ng total RNA using the iScript™ cDNA Synthesis kit (BIO-RAD) and diluted to an equivalent of 4 ng total RNA per µl. Gene primers for qPCR (Table S1) were designed using the primer NCBI BLAST-tool ([www.ncbi.nlm.nih.gov/tools/primer-blast/](http://www.ncbi.nlm.nih.gov/tools/primer-blast/)). RT-qPCR was performed using the amount of cDNA corresponding to 20 ng total RNA in a 20 µl mix containing 12.5 µl iQ™ SYBR® green Supermix (BIO-RAD) and 250 nM of both the forward and reverse primer. Reactions were performed on a CFX96™ Real-Time System (BIO-RAD) using a protocol of 40 cycles of 10 s at 95 °C and 30 s at 60 °C followed by a melt-curve analysis.

*References*

**Bode RF, Halitschke R, Kessler A. 2013.** Herbivore damage-induced production and specific anti-digestive function of serine and cysteine protease inhibitors in tall goldenrod, *Solidago altissima* L. (Asteraceae). *Planta* **237**:1287–1296.

**Bradford MM. 1976.** A rapid and sensitive method for the quantitation of microgram quantities of protein utilizing the principle of protein-dye binding. *Analytical Biochemistry* **72**:248–254.

**Kuhl C, Tautenhahn R, Bottcher C, Larson TR, Neumann S.** **2012** CAMERA: An integrated strategy for compound spectra extraction and annotation of liquid chromatography/mass spectrometry data sets. *Analytical Chemistry* **84**:283–289.

**Nakata M, Ohme-Takagi M. 2014.** Quantification of anthocyanin content. *Bioprotocol* **4**:e1098.

**Smith CA, Want EJ, O'Maille G, Abagyan R, Siuzdak G.** **2006** XCMS: Processing mass spectrometry data for metabolite profiling using nonlinear peak alignment, matching, and identification. *Analytical Chemistry* **78**:779–787.

**Thaler JS, Stout MJ, Karban R, Duffey SS. 1996.** Exogenous jasmonates simulate insect wounding in tomato plants (*Lycopersicon esculentum*) in the laboratory and field. *Journal of Chemical Ecology* **22**:1767–1781.

**Wintermans JF, Demots A. 1965.** Spectrophotometric characteristics of chlorophylls a and b and their pheophytins in ethanol. *Biochimica Et Biophysica Acta* **109**:448–453.
