# Supplementary material for: Slug Feeding Triggers Dynamic Metabolomic and Transcriptomic Responses Leading to Induced Resistance in Solanum dulcamara
Source: Front Plant Sci. 2020 Jun 18;11:803. doi: 10.3389/fpls.2020.00803 (PMC7314995; doi:10.3389/fpls.2020.00803)
Supplement: Supplementary file 2 [file Image_1.pdf]

## Supplementary figures

### Fig. S1

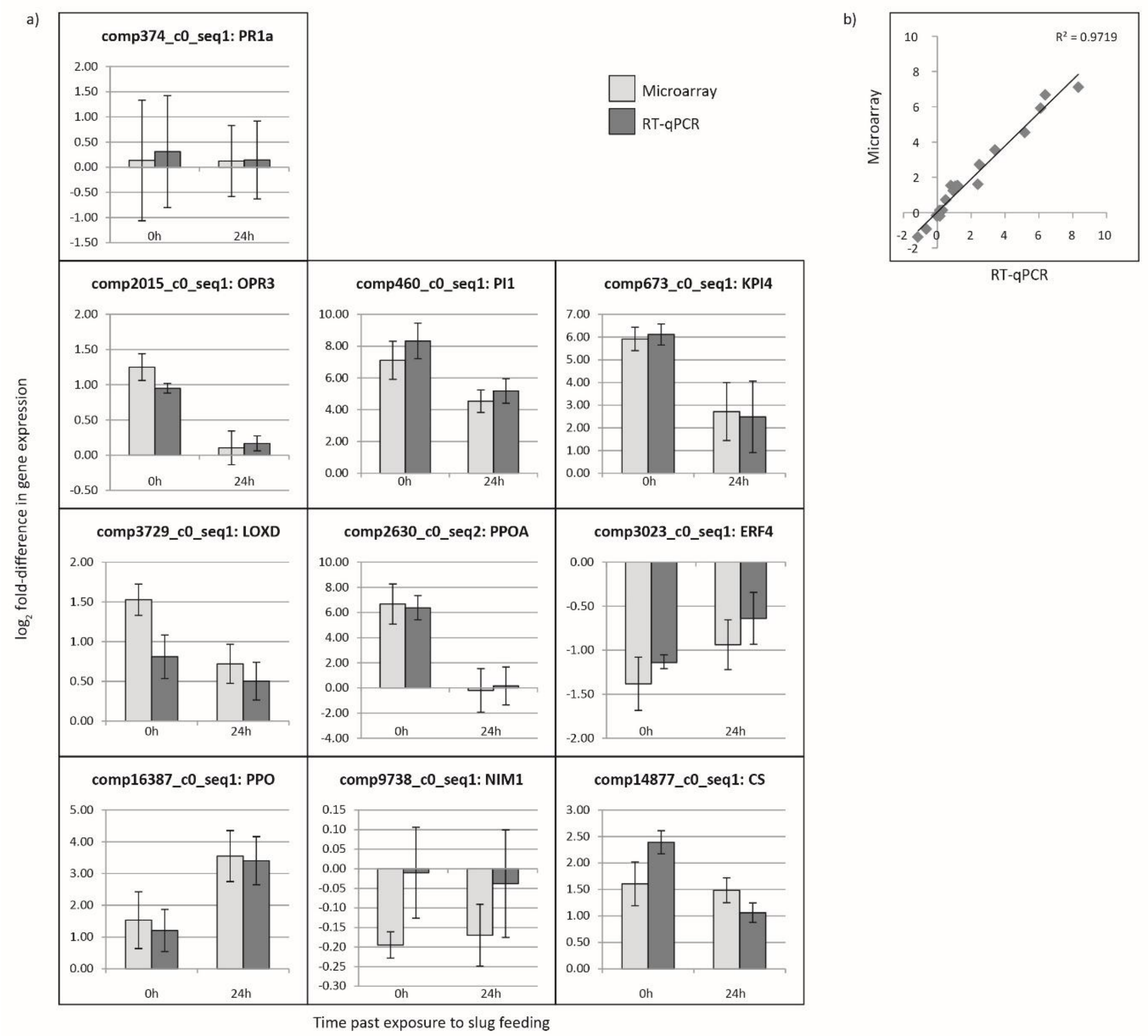


**Fig. S1** Validation of microarray analyses based on expression of 10 contigs as measured by qPCR (see Document S1) in *Solanum dulcamara* leaves upon feeding by the grey field slug (GFS, *Deroceras reticulatum*). **a**) mean (± SE) difference in expression relative to undamaged control samples directly (0h) after 24h of exposure to GFS or after an additional 24h without exposure to slug feeding. **b**) Correlation of contig expression as quantified by both methods

**Fig. S2**

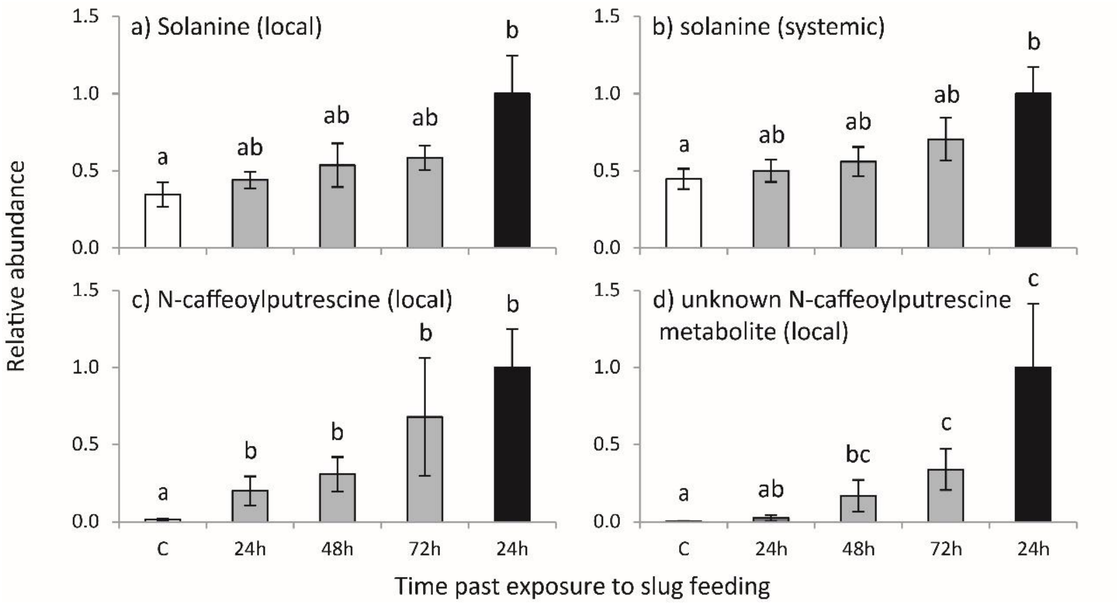


**Fig. S2** Induced responses in *Solanum dulcamara* leaves after feeding by the grey field slug (GFS, *Deroceras reticulatum*). Plants ($n$ = 6) were left undamaged (control treatment: C, white bar) or exposed to feeding by GFS for 24h (grey bars) or 72h (black bar). Samples were collected at 24h, 48h or 72h past the end of the treatment period (see also Fig. 1). Mean relative abundance (± SE) is shown of four defence metabolites that were selected from untargeted metabolomic analyses (Fig. 3). Treatment effects were further tested using One-Way ANOVA. **a**) Solasonine in local leaves (LGA3, F = 3.864, $P$ = 0.014); **b**) Solasonine in systemic leaves (SGA3, F = 3.283, $P$ = 0.027); **c**) N-caffeoylputrescine in local leaves (L005, F = 8.225, $P$ = <0.001); **d**) unknown N-caffeoylputrescine metabolite (*m/z* 347.2) in local leaves (L026, F = 12.360, $P$ = <0.001). Different letters over the bars indicate significant differences among treatments according to Tukey post-hoc test ($P$ <0.05).
